# PREX2 contributes to radiation resistance by inhibiting radiotherapy-induced tumor immunogenicity via cGAS/STING/IFNs pathway in colorectal cancer

**DOI:** 10.1186/s12916-024-03375-2

**Published:** 2024-04-12

**Authors:** Mingzhou Li, Jianbiao Xiao, Shasha Song, Fangyi Han, Hongling Liu, Yang Lin, Yunfei Ni, Sisi Zeng, Xin Zou, Jieqiong Wu, Feifei Wang, Shaowan Xu, You Liang, Peishuang Xu, Huirong Hong, Junfeng Qiu, Jianing Cao, Qin Zhu, Li Liang

**Affiliations:** 1 Department of Pathology, Nanfang Hospital, Southern Medical University, Guangzhou, 510515 People’s Republic of China; 2 Department of Pathology, School of Basic Medical Sciences, Southern Medical University, Guangzhou, 510515 People’s Republic of China; 3 Guangdong Provincial Key Laboratory of Molecular Tumor Pathology, Guangzhou, Guangdong People’s Republic of China; 4 Jinfeng Laboratory, Chongqing, 401329 People’s Republic of China; 5 Department of Pathology, Yantai Fushan People’s Hospital, Yantai, 265500 Shandong People’s Republic of China; 6 Yue Bei People’s Hospital Postdoctoral Innovation Practice Base, Southern Medical University, Guangzhou, 510515 People’s Republic of China

**Keywords:** Colorectal cancer, PREX2, Immunogenic cell death, Radioresistance, cGAS/STING/IFNs

## Abstract

**Background:**

Colorectal cancer (CRC) lacks established biomarkers or molecular targets for predicting or enhancing radiation response. Phosphatidylinositol-3,4,5-triphosphate-dependent Rac exchange factor 2 (PREX2) exhibits intricate implications in tumorigenesis and progression. Nevertheless, the precise role and underlying mechanisms of PREX2 in CRC radioresistance remain unclear.

**Methods:**

RNA-seq was employed to identify differentially expressed genes between radioresistant CRC cell lines and their parental counterparts. PREX2 expression was scrutinized using Western blotting, real-time PCR, and immunohistochemistry. The radioresistant role of PREX2 was assessed through in vitro colony formation assay, apoptosis assay, comet assay, and in vivo xenograft tumor models. The mechanism of PREX2 was elucidated using RNA-seq and Western blotting. Finally, a PREX2 small-molecule inhibitor, designated PREX-in1, was utilized to enhance the efficacy of ionizing radiation (IR) therapy in CRC mouse models.

**Results:**

PREX2 emerged as the most significantly upregulated gene in radioresistant CRC cells. It augmented the radioresistant capacity of CRC cells and demonstrated potential as a marker for predicting radioresistance efficacy. Mechanistically, PREX2 facilitated DNA repair by upregulating DNA-PKcs, suppressing radiation-induced immunogenic cell death, and impeding CD8^+^ T cell infiltration through the cGAS/STING/IFNs pathway. In vivo, the blockade of PREX2 heightened the efficacy of IR therapy.

**Conclusions:**

PREX2 assumes a pivotal role in CRC radiation resistance by inhibiting the cGAS/STING/IFNs pathway, presenting itself as a potential radioresistant biomarker and therapeutic target for effectively overcoming radioresistance in CRC.

**Supplementary Information:**

The online version contains supplementary material available at 10.1186/s12916-024-03375-2.

## Background

Colorectal cancer (CRC) ranks as the third most frequently diagnosed cancer and the second leading cause of cancer-related mortality globally [1]. The standard treatment for locally advanced CRC involves neoadjuvant chemoradiotherapy (nCRT) followed by surgery [2]. Approximately 15−20% of CRC patients achieve a pathological complete response (pCR) after nCRT [3, 4]. Despite this, a majority of patients do not derive the benefits of nCRT. Specifically, preoperative radiotherapy may lead to disease progression in patients with limited response to radiotherapy, consequently delaying surgical intervention. Moreover, no identified pathways of specific genes have been successfully targeted in clinical settings to enhance radiation sensitivity. Therefore, it is imperative to elucidate the molecular mechanisms related to radioresistance and identify novel biomarkers to screen CRC patients sensitive to preoperative radiotherapy. This endeavor may offer new avenues to ameliorate the poor prognosis of CRC patients.

While radiotherapy holds the potential to induce antitumor immunity, its molecular mechanisms remain inadequately understood [5]. DNA damage, with double-strand breaks (DSBs) being the most lethal form, constitutes the major mechanism contributing to radiation-induced cell killing. The sensitivity of cancer cells to radiation largely hinges on their ability to recognize and respond to DSBs [6]. Previous research indicates that ionizing radiation (IR) induces DSBs, and DNA fragments leak through the damaged nuclear membrane, augmenting the accumulation of double-stranded DNA (dsDNA) in the cytoplasm [7]. These dsDNAs, detected by cyclic GMP-AMP synthase (cGAS), initiate the cGAS/stimulator of interferon genes (STING) signaling pathway, leading to subsequent type I interferon (IFN-I) signaling. This cascade promotes the activation of CD8^+^ cytotoxic T cell-mediated cancer destruction [8, 9]. Type I IFNs have been shown to play a critical role in bridging DNA damage in tumor cells and antitumor adaptive immunity following radiation [10]. Notably, IFN-I production is among the inducing factors of immunogenic cell death (ICD) [11, 12]. ICD, a regulatory cell death (RCD) induced by cellular stress accompanied by exposure of damage-associated molecular patterns (DAMPs), includes events such as calreticulin (CRT) exposure to the cell surface, high mobility group box 1 protein (HMGB1) and adenosine-5′-triphosphate (ATP) release, and production of IFN-I [11, 12]. ICD enhances the activation of dendritic cells (DCs), ultimately amplifying the CD8^+^ T cell-triggered immune responses. Thus, activation of the cGAS/STING/IFN-I pathway emerges as a potential strategy to enhance IR sensitivity. However, studies focusing on the precise molecular mechanisms by which radiotherapy affects the cGAS/STING/IFN-I pathway are still limited.

In this context, an analysis of RNA-sequencing (RNA-seq) data comparing the IR-resistant cell line IR-SW480 with the IR-sensitive cell line SW480 led to the identification of a gene, phosphatidylinositol-3,4,5-triphosphate-dependent Rac exchange factor 2 (PREX2, also known as P-Rex2a), exhibiting high expression in IR-SW480 and a positive correlation with the radioresistance of CRC. PREX2 is an 182 kDa protein sensitive to phosphoinositide 3-kinase (PI3K) and belongs to the Rac guanine nucleotide exchange factor family (RacGEFs) [13]. Recognized as an oncogene, PREX2 displays abnormal expression in various tumors, with mutations or abnormal expression observed in melanoma [14], breast [15], ovarian [15], prostatic [15], hepatocellular carcinoma [16], and pancreatic cancer [17]. By inhibiting the activity of phosphatase and tensin homolog (PTEN), PREX2 regulates the downstream PI3K signaling pathway [18] and is identified as a known driver gene in CRC [19]. Despite numerous studies reporting the carcinogenic effect of PREX2, little is known about its role and regulatory mechanisms in cancer radiotherapy sensitivity

In the present study, we aimed to elucidate the expression pattern and biological role of PREX2 in CRC radioresistance. Our findings revealed that PREX2 overexpression significantly diminished CRC radiotherapy sensitivity both in vitro and in vivo. Additionally, PREX2 promoted DNA double-strand break repair by upregulating DNA-dependent protein kinase catalytic subunit (DNA-PKcs) and inhibiting radiation-induced immunogenic cell death and CD8^+^ T cell infiltration through the cGAS/STING/IFNs pathway. Targeting PREX2 with the small molecule P-Rex inhibitor 1 (PREX-in1) substantially heightened the efficacy of CRC IR therapy and increased CD8^+^ T cell infiltration. Our data demonstrate that PREX2 serves as a novel biomarker for CRC sensitivity to preoperative radiotherapy and represents a therapeutic target for radiotherapy-resistant CRC patients.

## Methods

### Patients and tissues

A total of 52 pathological specimens were obtained from CRC patients between January 2017 and January 2021 at the Department of Pathology, Nanfang Hospital, Southern Medical University. All cases used in the current study were collected from pre-treatment colonoscopy specimens of patients with nCRT and without any antitumor therapies, including surgery, targeted therapy, biological therapy, and immunotherapy. The 8th edition of the American Joint Council on Cancer (AJCC) tumor regression grading system (TRG) was used for the pathological analysis of tumor specimens after nCRT to evaluate the therapeutic effect [20]. TRG 0 represents complete response (absence of residual cancer cells), TRG 1 represents moderate response (only small clusters or single cancer cells remain), TRG 2 represents minimal response (residual cancer is present with predominant fibrosis), and TRG 3 represents poor response (almost no fibrosis and a large amount of residual cancer). We identified patients with TRG 0 and TRG 1 as radiotherapy responders and those with TRG 2 and TRG 3 as radiotherapy-resistant patients. All specimens were collected and analyzed after obtaining written informed consent from the patients. This study was approved by the Ethics Committee of Nanfang Hospital of Southern Medical University (Guangzhou, China) (Approval No. NFEC-2023-026).

### Cell culture

The cell lines used in this study, including the human CRC cell lines SW480, CaCo2, HCT15, HCT116, SW620, HT29, LoVo, RKO, and mouse CRC cell line MC38, were purchased from the American Type Culture Collection (ATCC) and were stored in the Department of Pathology of Southern Medical University. SW480 and SW620 cells were cultured in Leiboviz’s L-15 medium (Gibco) supplemented with 10% fetal bovine serum (FBS) (Gibco). CaCo2, LoVo, HT29, and MC38 cells were cultured in DMEM (Gibco) supplemented with 10% FBS. HCT15 and RKO cells were cultured in RPMI 1640 medium (Gibco) supplemented with 10% fetal bovine serum (FBS). HCT116 cells were cultured in McCoy’s 5A medium (Gibco) supplemented with 10% fetal bovine serum (FBS). All cells were cultured at 37 °C in a 5% CO_2_ atmosphere.

### Ionizing radiation and construction of IR-resistant SW480 and CMT93 cell lines

The cells or mouse tumors were irradiated with defined doses of ionizing radiation using 6 MV X-rays from linear accelerators (Varian 2300EX, Varian, 58 Palo Alto, CA, USA). To construct IR-resistant SW480 cell lines, the SW480 cells were exposed to radiation doses of 4 Gy with a dose rate of 5 Gy/min once daily at 24 h intervals, and the culture was continued after the end of the irradiation; the cycle lasted 8 times. The irradiation can reach 40 Gy, which is the total planned dose of clinical radiotherapy. The cells that survived all the IR cycles were named IR-SW480 and IR-CMT93. The surviving cells were evaluated for radiosensitivity using clonogenic survival assays.

### Plasmids and construction of stable cell lines

PREX2 was constructed by cloning the PCR-amplified full-length human PREX2 cDNA into pEZ-Lv201. The pLKD-Puro vector was used to clone short hairpin RNA (shRNA) targeting PREX2. The shRNA sequences targeting PREX2 were obtained from the shRNA sequence prediction website portal (Additional file 1: Table S1). pEZ-Lv201 and pLKD-Puro were purchased from Addgene (Cambridge, MA). The PREX2 overexpressed or knocked down lentivirus was generated by co-transfecting 293T cells with two packing vectors, psPAX and pMD2G, and Lipofectamine 2000 (Invitrogen), and then collecting the supernatants of 293T culture medium after 48 h and filtering through 0.45-mm filters (Millipore, Temecula, CA, USA), and they were concentrated. The expression of PREX2 was detected using real-time PCR and Western blotting.

### Enzyme-linked immunosorbent assay (ELISA)

The amount of HMGB1 protein in the supernatant was determined using human HMGB1-specific ELISA kits (Cusabio, #CSB-E08223h) according to the manufacturer’s instructions. The experiment was performed three times with three replicates in each experiment.

### Extracellular ATP assessment

After treatment, the cell culture medium was collected, and the supernatant was processed by centrifugation. ATP levels were determined by using the reagent kit (Beyotime, #S0027) and luciferin-based assay for ATP concentration. The experiment was performed three times with three replicates in each experiment.

### RNA extraction and real-time PCR

RNA isolation was performed as described previously [21]. Total RNA was extracted from cell lines using TRIzol (Invitrogen, USA), quantified by measuring the absorbance at 260 nm, and then reverse transcribed into cDNAs according to the manufacturer’s instructions.

Real-time quantitative reverse transcription PCR (RT-qPCR) was performed at least three times in triplicate using SYBR Green mix (Vazyme, Q711-02) and the ABI PRISM 7500 Sequence Detection System (Applied Biosystems, USA). Target gene expression was normalized to the geometric mean of the housekeeping gene GAPDH and was calculated using the 2^−ΔΔCT^ method. Primer Express was used to design the real-time PCR primers, and the primer sequences for amplification are shown in Additional file 1: Table S1. The experiment was performed three times with three replicates in each experiment.

### Western blot

Western blotting was performed as previously described [22]. The antibodies used are listed in Additional file 2: Table S2.

### Immunohistochemistry

Immunohistochemical (IHC) staining was performed as previously described using specific antibodies [22]. The antibodies used are listed in Additional file 2: Table S2. Two observers independently reviewed and scored independently by two observers. Staining intensity was graded according to the following criteria: 0 (no staining), 1 (weak staining or light yellow), 2 (moderate staining or yellow-brown), and 3 (strong staining or brown). The proportion of tumor cells was scored as follows: 0 (no positive tumor cells), 1 (< 25% positive tumor cells), 2 (26–50% positive tumor cells), 3 (55–75% positive tumor cells), and 4 (> 75% positive tumor cells). The staining index was calculated as the staining intensity score × proportion of positive tumor cells. Optimal cut-off values were identified: a staining index ≥ 6 was used to define tumors with high PREX2 expression, and an index < 6 was used to define tumors with low PREX2 expression.

### Immunofluorescence

The cells were grown on coverslips after irradiation with 4 Gy. Paraformaldehyde (4%) was used to fix the cells for approximately 10 min, followed by incubation with 0.1% Triton X-100 for approximately 30 min. All samples were placed in a 1% BSA solution (dilution ratio; see instructions) and closed on a horizontal shaker for 30 min. The primary antibody solution and incubated overnight at 4 °C. The corresponding fluorescent dye-conjugated secondary antibody was added and incubated in the dark for 1 h, and 10 μL DAPI was added for 15 min. Finally, images were collected using fluorescence microscopy or confocal microscopy. The antibodies used are listed in Additional file 2: Table S2. The experiment was performed three times with three replicates in each experiment.

### Radiation clonogenic assay

Cells were seeded on six-well plates at a density of 4 × 10^2^, 8 × 10^2^, 1 × 10^3^, 5 × 10^3^, and 8 × 10^3^ cells per well and exposed to IR at doses of 0 Gy, 2 Gy, 4 Gy, 6 Gy, and 8 Gy respectively. After incubation for 14 d at 37 °C, the plated cells were washed with PBS, fixed with 4% paraformaldehyde for 30 min, and stained with crystal violet for 20 min. Colonies (≥ 50 cells/colony) were counted under a dissecting microscope. The surviving fraction (SF) curve was revised via a multi-target single-hit model with the following formula “SF = 1 − (1 − e^−D/D0^) ^N^”. The experiment was performed three times with three replicates in each experiment.

### Comet assay

For the comet assay, single-cell suspensions were prepared and collected for the experiments. After gel preparation, the gels were poured into a pre-chilled lysis buffer, split at 4 °C for 2 h. The slides were removed, washed, and placed in anhydrous ethanol for 5 min after electrophoresis. After adding PI staining solution to each slide and using 515–560 nm excitation light for fluorescence microscopy, DNA images of PI samples were red. Finally, nuclear DNA and migrated DNA were observed using Open Comet software to measure nuclear DNA diameter and DNA migration length. The experiment was performed three times with three replicates in each experiment.

### Flow cytometry

For apoptosis analysis, the cells were irradiated and collected at 0 h and 48 h cells to prepare single-cell suspensions. The cells were then operated according to the APC Annexin V/PI Apoptosis Detection Kit (BioLegend #640932). For cell cycle analysis, the harvested cells were incubated with DNA staining and permeabilization solution (protected from light, room temperature, 30 min) and then analyzed by flow cytometry. To determine the expression of calreticulin, the cells were stained with the respective primary antibodies, followed by Alexa Fluor 488 anti-mouse IgG. The experiment was performed three times with three replicates in each experiment.

To investigate immune cell infiltration, we first isolated tumor-infiltrating lymphocytes (TILs) by Percoll. The TILs were incubated with antibodies conjugated with fluorescent biotin specific for CD45, CD4, and CD8A for 30 min on ice. After incubation, the cells were resuspended and analyzed using flow cytometry. The antibodies used are listed in Additional file 2: Table S2.

### RNA-seq and data analysis

Total RNA was extracted using the TRIzol reagent kit (Invitrogen, Carlsbad, CA, USA) according to the manufacturer’s protocol. RNA quality was assessed on an Agilent 2100 Bioanalyzer (Agilent Technologies, Palo Alto, CA, USA) and verified by RNase-free agarose gel electrophoresis. After total RNA was extracted, eukaryotic mRNA was enriched with Oligo (dT) beads, fragmented into short fragments using fragmentation buffer, and reverse transcribed into cDNA using NEBNext Ultra RNA Library Prep Kit for Illumina (NEB #7530, New England Biolabs, Ipswich, MA, USA). The purified double-stranded cDNA fragments were end-repaired and a base was added and ligated to Illumina sequencing adapters. The ligation reaction was purified using AMPure XP Beads (1.0X). The ligation products were size-selected by agarose gel electrophoresis, PCR-amplified, and sequenced using an Illumina NovaSeq6000 by Gene Denovo Biotechnology Co. (Guangzhou, China). Quality control of raw reads was performed using fastp (version 0.18.0) to obtain clean reads. An index of the reference genome was constructed, and paired-end clean reads were aligned to the reference genome using HISAT2.2.4 with the parameter “-rna-strandness RF” and default settings. The aligned reads for each sample were assembled using StringTie v1.3.1 in a reference-based approach. Fragment per kilobase of transcript per million mapped reads values were calculated for each transcription region to quantify expression abundance and variations, utilizing the RSEM software. Differentially expressed genes (DEGs) within SW480 and IR-SW480 groups, or between IR-SW480 cells and PREX2 knockdown IR-SW480 cells, were identified with an adjusted *p*-value of < 0.05 using the R package “limma.” The “ggplot2” package facilitated the creation of a volcano plot illustrating the DEGs, while the top 50 DEGs’ expressions were visualized in a heatmap generated by the “pheatmap” package. To further explore the associated signaling pathways of PREX2, the Gene Set Enrichment Analyses (GSEA) software was employed to identify differentially enriched pathways between IR-SW480 and PREX2 knockdown IR-SW480 groups. Additionally, Gene Ontology (GO) function enrichment analysis was conducted based on DEGs between IR-SW480 cells and PREX2 knockdown IR-SW480 cells.

### Animal model

Animal studies received approval from the Committee on the Ethics of Animal Experiments of Southern Medical University (Approval No. L2019023). Female C57/B6J mice (5–6 weeks) and nude mice (4–6 weeks old) were procured from the Guangdong Animal Center. For the xenograft subcutaneous tumor model, 2 × 10^6^ cells/100 μL of tumor cells were injected subcutaneously into the rump dorsum of nude mice. CMT93 (1 × 10^6^ cells/100 μL) was subcutaneously injected into the flanks of C57/B6J mice. Upon reaching a tumor volume of 100 mm^3^, they were irradiated with 8 Gy three times in total, with an irradiation interval of 1 day. The mice were anesthetized with pentobarbital of 50 mg/kg (intraperitoneally). After euthanasia by cervical dislocation within 8 weeks, tumor tissues underwent formaldehyde fixation, paraffin-embedding, and 3-mm sections were cut for H&E and IHC staining.

For the subcutaneous injection of MC38 and IR-CMT93 cells (1 × 10^6^ cells/100 μL) into C57/B6J mice. Mice were randomly assigned to groups 2 weeks before receiving IR treatment (8 Gy ×3 fractions) at 2-day intervals. Small-molecule inhibitors of PREX2 (PREX-in1, 0.8 mg/kg; Specs, The Netherlands) were intraperitoneally injected 3 h before IR treatment. Tumor volume (*V*) was monitored every 2 days by measuring the short axis (*W*) and the long axis (*L*) of the xenograft tumor and calculated with the following formula: *V* = (*L* × *W*^2^)/2. At the end of the experiment, all mice were sacrificed by cervical dislocation, tumor tissues were dissected, photographed, and subjected to flow cytometry for tumor-infiltrating lymphocytes or formaldehyde fixation for IHC analyses.

### Bioinformatics analysis

To investigate the correlation between PREX2 and radiosensitivity, gene expression profiles and clinical information were obtained from the public Gene Expression Omnibus (GEO) database (https://www.ncbi.nlm.nih.gov/geo/) and the Cancer Genome Atlas (TCGA) databases (https://www.cancer.gov/ccg/research/genome-sequencing/tcga). Our study utilized two GEO cohorts (GSE145037 and GSE150082). PREX2 expression was analyzed based on the patient’s sensitivity to radiotherapy. The radiosensitivity index (RSI) was calculated according to the reported methods [23], RSI = − 0.0098009 × AR + 0.0128283 × Jun + 0.0254552 × STAT1-0.0017589 × PRKCB-0.0038171 × RELA + 0.1070213 × ABL1-0.0002509 × SUMO-0.0092431 × CDK1-0.0204469 × HDAC1-0.0441683 × IRF1. The radiosensitivity index (RSI) was calculated, and patients were divided into RSI-low and RSI-high groups according to the median RSI value in TCGA-COADREAD. Kaplan–Meier method and log-rank method were employed to assess the relationship between PREX2 expression and progression-free interval by using the R package “survival.” The algorithm determined the split point where the *p*-value was minimal.

To explore the correlation between PREX2 and immune infiltration in CRC, the Estimating the Proportions of Immune and Cancer Cell (EPIC) algorithm evaluated the immunity scores for each TCGA-COADREAD patient [24].

### Statistical analysis

Data statistics were performed using the IBM SPSS Statistics 22 version software. The GraphPad Prism 9 statistical software was used for data processing. Correlation coefficients were determined using Spearman’s rank correlation test. Unpaired two-tailed Student’s *t*-test was used to compare the two groups. Two-way ANOVA analysis of variance was used to compare the two groups in the animal experiments. For comparison of more than two groups, *p*-values were calculated using one-way ANOVA. Survival curves were plotted using the Kaplan–Meier method and compared by the log-rank test. *p < 0.05; **p < 0.01; ***p < 0.001; ns, not significant.

## Results

### PREX2 as a novel predictor of poor response to radiotherapy in CRC

**Fig. 1:**
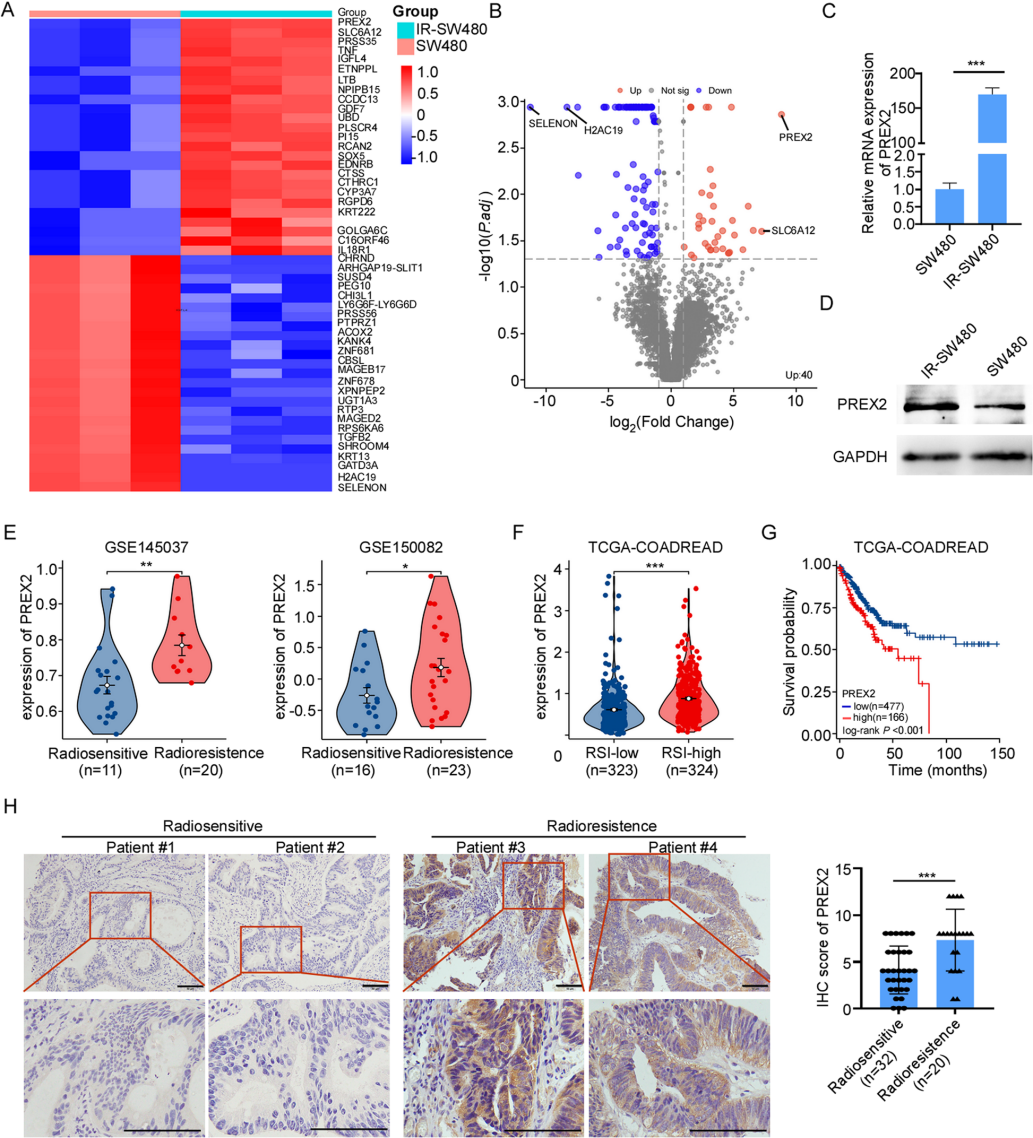
PREX2 acts as a radioresistant gene in CRC. **A**, **B** heat maps and volcanic maps showed significant differential genes after RNA-seq screening between IR-resistant cell line IR-SW480 and IR-sensitive cell line SW480. **C**, **D** qRT-PCR and Western blot analysis were conducted to determine the expression of PREX2 in IR-SW480 and SW480. **E** The analysis of PREX2 expression in radiotherapy-resistant CRCs compared with radiotherapy-sensitive CRCs in CRC microarray profile (GES145037 responder: 0.674 ± 0.109, non-responder: 0.784 ± 0.096, *p* = 0.0084; GSE1150082 responder: − 0.261 ± 0.125, non-responder: 0.180 ± 0.143, *p* = 0.0345). **F** RSI was calculated using the data from the TCGA database and the expression of PREX2 in the low RSI group and high RSI group was analyzed. **G** Kaplan–Meier analysis of progression-free interval in all patients with CRC according to PREX2 expression in TCGA dataset (log-rank test *p* < 0.001). **H** The expressions of PREX2 protein in specimens, including 54 CRC tissue specimens, were examined by IHC. Representative IHC images (left) and correlation analysis (right) were shown. Scale bar: 50 μm. *p < 0.05, **p < 0.01, ***p < 0.001

In pursuit of a deeper comprehension of cellular mechanisms underpinning radiation resistance, we initially established and validated a radiotherapy-resistant cell line, IR-SW480 (Additional file 3: Fig. S1A-C). Subsequently, RNA-seq was conducted between the IR-resistant cell line IR-SW480 and the IR-sensitive cell line SW480. Differentially expressed gene (DEGs) analysis unveiled a set of genes exhibiting substantial differences in expression (Fig. 1A, Additional file 4: Table S3). Notably, among these DEGs, PREX2 and SLC6A12 (solute carrier family 6 member 12) emerged as the top two upregulated genes, while SELENON (selenoprotein N) and H2AC19 (H2A clustered histone 19) were identified as the top two downregulated genes in response to radiotherapy resistance (Fig. 1A, B). Given the high expression of PREX2 in the IR-sensitive cell line SW480 and the significance of this difference, our focus in this study centered on PREX2. Additionally, RT-qPCR and Western blotting validate the expression of PREX2 in these two cell lines, showcasing a substantial upregulation of PREX2 in IR-SW480 cells compared to SW480 (Fig. 1C, D).

Further investigation into the correlation between PREX2 and radiosensitivity employed clinical data extracted from the GEO and TCGA databases. In accordance with our previous findings, PREX2 expression demonstrated a significant upregulation in radiotherapy-resistant CRC tissues (GSE145037 and GSE150082) (Fig. 1E). The radiosensitivity index, a genome-based mode estimating the intrinsic radiosensitivity of tumors inversely correlated with radiosensitivity [23], indicated that PREX2 expression in RSI-high CRC exceeded that in RSI-low CRC within the TCGA-READCOAD datasets (Fig. 1F). Kaplan–Meier survival analysis of TCGA-READCOAD datasets revealed a markedly poorer progression-free interval in CRC patients with high PREX2 expression compared to those with low PREX2 expression (*p* = 0.0006, Fig. 1G).

Furthermore, PREX2 expression was scrutinized through IHC in an extended cohort of 52 paraffin-embedded CRC samples. The results demonstrated heightened PREX2 expression in radiotherapy-resistant tissues (Fig. 1H), correlating significantly with the TRG classification. However, PREX2 expression did not exhibit correlation with advanced TNM classification, cancer histologic grade, or position (Additional file 5: Table S4). These findings collectively suggest a strong association between PREX2 and radioresistance in CRC.

### PREX2 enhances the radiation resistance of CRC cells in vitro and in vivo

**Fig. 2:**
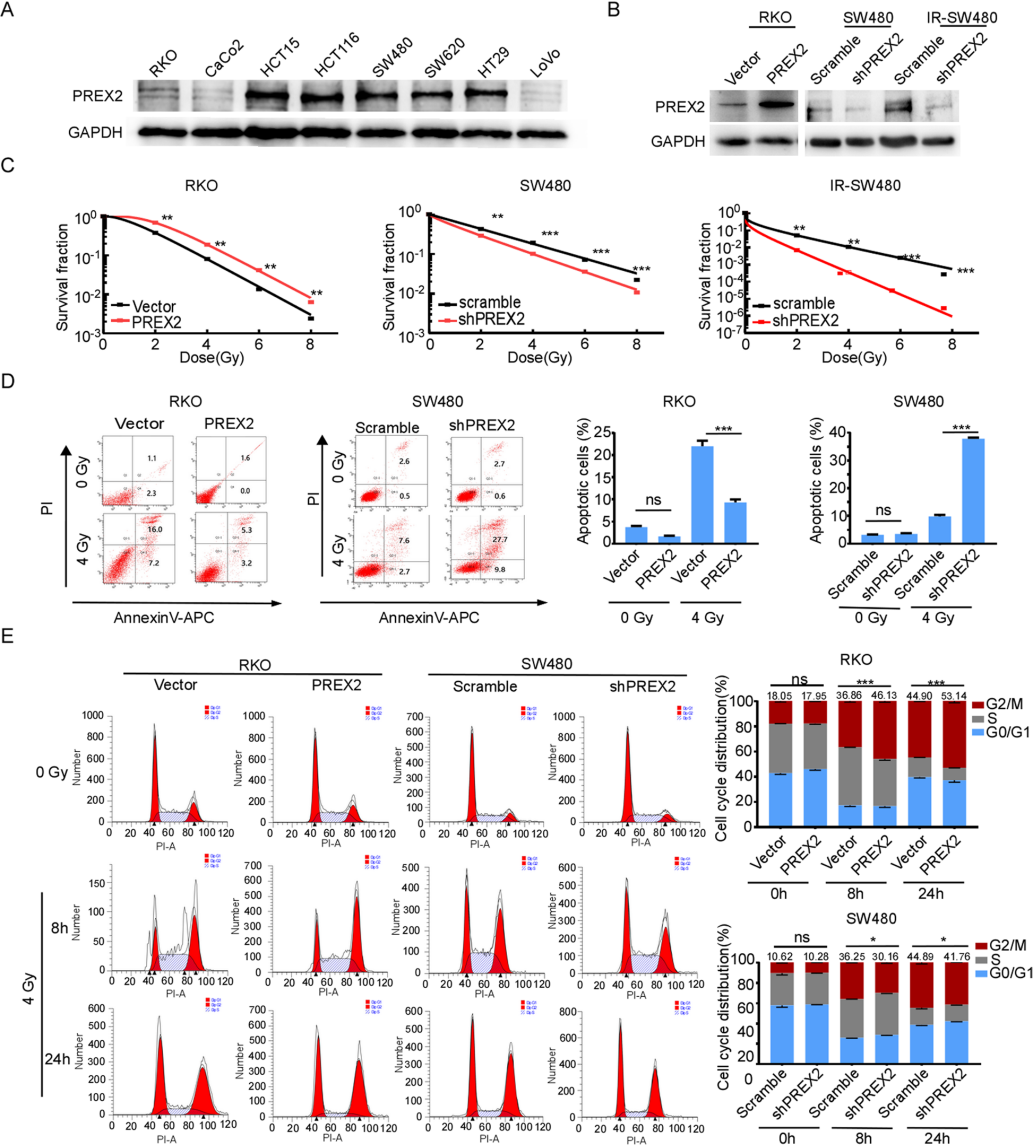
PREX2 promotes the radiation resistance of CRC cells in vitro. **A** A basal level of PREX2 was determined using Western blot analysis in several CRC cell lines (including RKO, CaCo2, HCT15, HCT116, SW480, SW620, HT29, and LoVo). **B** Stable overexpression and interference cell lines were detected by Western blotting. **C** Survival fraction with multi-target single-hit model. **D** Flow cytometry was performed to detect apoptosis of stable overexpression and interference cell lines with or without treatment with 4 Gy radiation. **E** Cell cycle progression was analyzed by flow cytometry. ns not significant, *p < 0.05, **p < 0.01, ***p < 0.001

To elucidate the biological functions of PREX2 in the radioresistance of CRC, the endogenous expression of PREX2 in CRC cell lines was assessed via Western blotting (Fig. 2A). Subsequently, PREX2 overexpression was induced in RKO cell lines with low endogenous expression, while PREX2 knockdown was implemented in SW480 and IR-SW480 cell lines with high endogenous expression (Fig. 2B, Additional file 6: Fig. S2A). Radiation clonogenic assays showed that PREX2 overexpression induced radioresistance in RKO cells, whereas PREX2 depletion heightened radiosensitivity in SW480 and IR-SW480 cells (Fig. 2C, Additional file 6: Fig. S2B).

Further assessment included the examination of changes in cell apoptosis using flow cytometry with Annexin V-APC/PI staining. PREX2 overexpression notably reduced the rate of apoptosis in RKO cells treated with 4 Gy radiation, while PREX2 knockdown significantly increased the rate of apoptosis in SW480 and IR-SW480 cells (Fig. 2D, Additional file 6: Fig. S2C). Flow cytometry was also employed to evaluate the effects of PREX2 on cell cycle distribution. G2/M phase arrest was more pronounced in PREX2 overexpressed RKO cells compared to control cells both at 8 h and 24 h after 4 Gy radiation but decreased under PREX2 knockdown conditions (Fig. 2E, Additional file 6: Fig. S2D). These findings indicate the involvement of PREX2 in the activation of the cell cycle checkpoint.

**Fig. 3:**
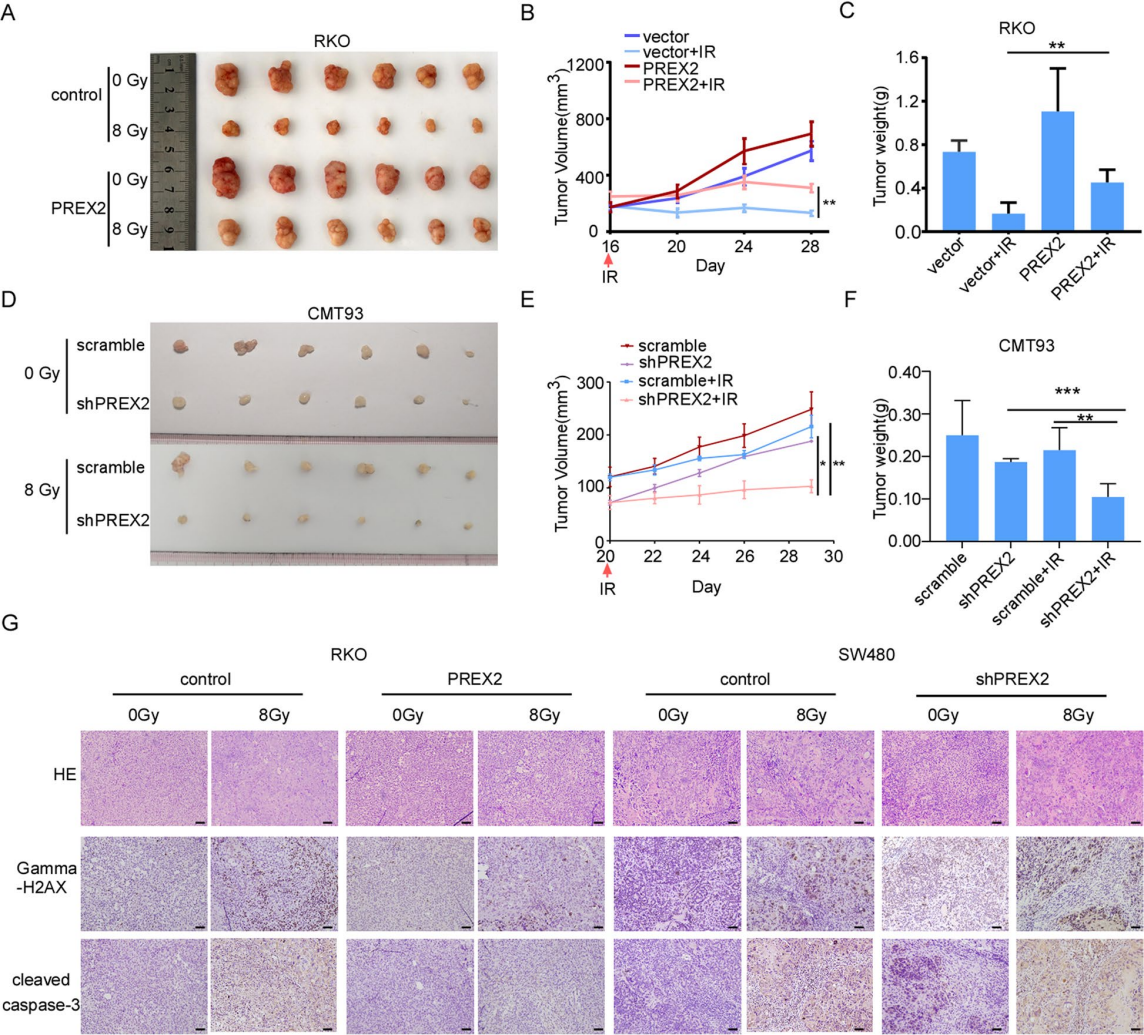
PREX2 inhibits radiosensitization of CRC in vivo. **A**–**C** Subcutaneous tumor formation in nude mice was established with PREX2-overexpressing or control RKO cells and treated with or without IR (*n* = 6/group). **A** The representative image of tumors derived from xenograft mice. **B**, **C** The volume and weight of tumors were monitored. Data were shown as means ± SEM. **D**–**F** Subcutaneous tumor formation in C57/B6J mice was established with PREX2- knockdown or control CMT93 cells and treated with or without IR (*n* = 6/group). **D** Representative images of each group were photographed at the end of the experiment. **E**, **F** The volume and weight of tumors were monitored. Data were shown as means ± SEM. **G** Subcutaneous tumors in nude mice were stained with hematoxylin and eosin (H&E, top). The representative IHC images of cleaved-caspase 3 and γH2AX expression in xenograft tumors were shown (below). Scale bar: 100 μm. *p < 0.05, **p < 0.01,***p < 0.001

Subsequently, we explored whether the observed phenomenon can be replicated in vivo. The overexpression of PREX2 resulted in a higher rate of tumor growth than the control group, causing a less pronounced reduction in xenograft volume post-IR (Fig. 3A–C). Conversely, PREX2 knockdown further suppressed tumor growth and reduced tumor volume in both C57/B6J and nude mice after radiotherapy (Fig. 3D–F, Additional file 6: Fig. S2E-H). Xenografts were analyzed for apoptosis and DNA repair ability through immunostaining for cleaved caspase-3 and phosphorylated histone H2AX (γH2AX). IHC staining demonstrated that overexpression of PREX2 significantly decreased, while knockdown of PREX2 significantly increased the expression of cleaved caspase-3 and γH2AX in mice that received irradiation (Fig. 3G). These results collectively underscore the role of PREX2 in promoting radioresistance in CRC, both in vitro and in vivo.

### PREX2 promotes DNA repair by increasing DNA-PKcs expression in CRC cells

**Fig. 4:**
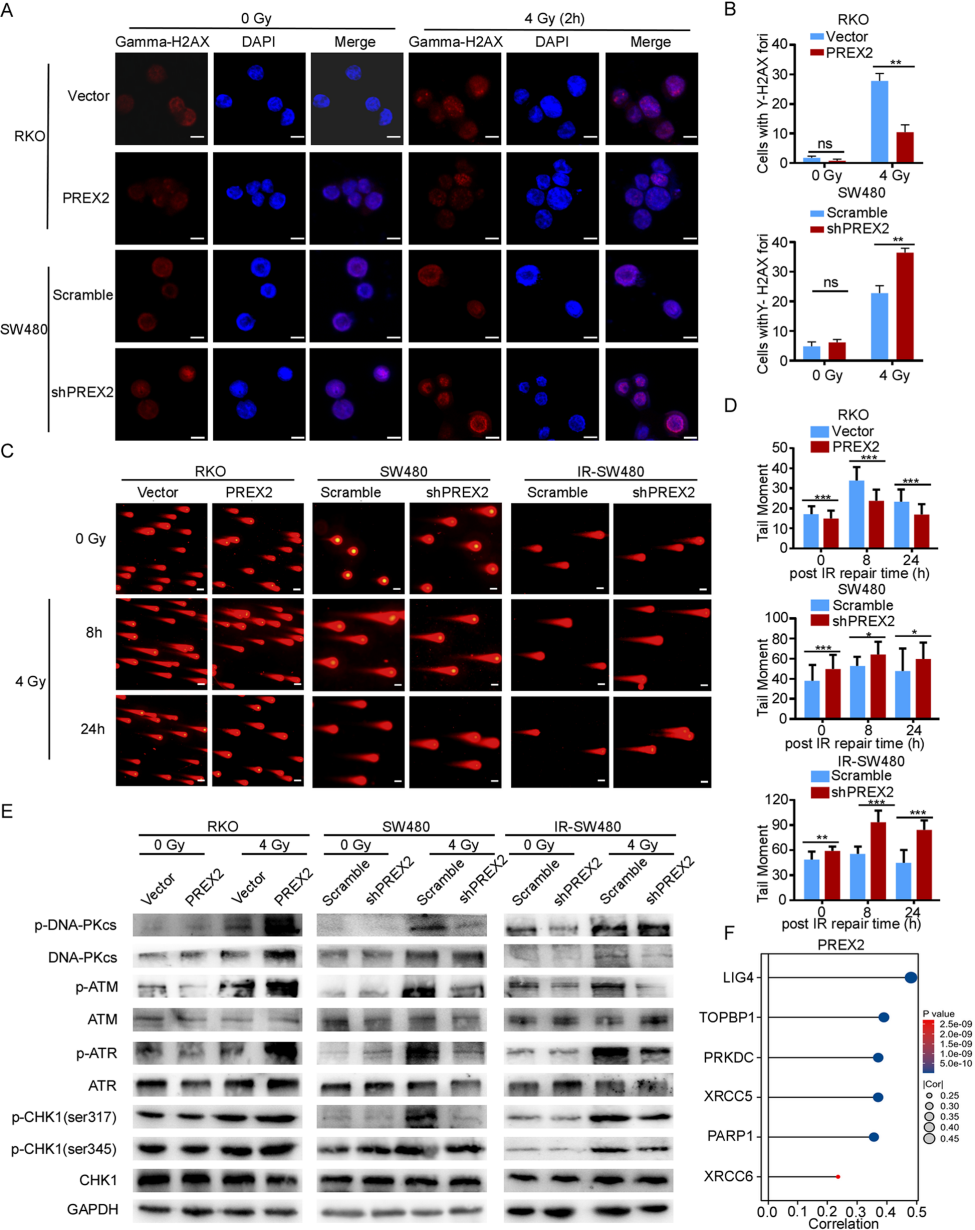
PREX2 modulated radiation-induced DNA damage response by regulating NHEJ repair. **A** Representative fluorescence images of γH2AX staining. Scale bar: 10 μm. **B** Quantification of γ-H2AX immunostaining in PREX2 overexpressed RKO cells and PREX2 knocked down SW480 cells with or without 4 Gy radiation. **C**, **D** The comet assay of stable overexpression and interference cell lines after IR treatment (scale bar: 50 μm, **C**) and the statistics result of quantification of tail moment (**D**). **E** Western blot detection of the expression levels of DNA-PKc, p-DNA-PKc, p-ATM, ATM, p-ATR, ATR, p-CHK1, and CHK1 in stable overexpression and interference cell lines with or without 4 Gy radiation. **F** Bioinformatics analysis of the correlation between PREX2 and genes related to NHEJ pathway (XRCC6, XRCC5, LIG4, PARP1, TOPBP1 and PRKDC) in TCGA-CRC. ns, not significant, *p < 0.05, **p < 0.01, ***p < 0.001

The primary mechanism of tumor radiotherapy revolves around the induction of DNA damage. Existing studies have underscored that the repair of damaged DNA in cancer cells dictates radiosensitivity [25, 26]. To discern whether PREX2 influences in DNA damage response (DDR), we initially assessed the impact of PREX2 on IR-induced DNA damage during radiotherapy. The levels of γH2AX, indicative of double-strand breaks (DSBs), were evaluated using an immunofluorescence assay. PREX2 overexpression notably reduced the number of γ-H2AX foci in RKO cell lines 2 h after 4 Gy radiation (Fig. 4A, B). Conversely, PREX2 knockdown resulted in an increased number of γ-H2AX foci compared to control cells at the same time point (Fig. 4A, B). Subsequently, a comet assay was conducted, revealing heightened DNA damage at 8 h and 24 h in irradiated PREX2 knockdown cells and diminished damage in PREX2 overexpressed RKO cells after IR (Fig. 4C, D). These findings suggest that the absence of PREX2 leads to unrepaired DNA damage post-IR.

Activation of DDR is known to be initiated by the phosphorylation of DNA-PKcs, ATM (ataxia telangiectasia mutated), ATR (ATM and Rad3-relate), and CHK1 (checkpoint kinase 1) as well as their substrates. To further elucidate the potential correlation between PREX2 and DDR in human CRC, the protein levels of key genes associated with DNA damage and repair were examined via immunoblotting. The results indicated that PREX2 deficiency reduced DNA-PKcs levels (Fig. 4E). Furthermore, in PREX2 knocked down SW480 and IR-SW480 cell lines, the phosphorylation of DNA-PKcs at S2056, ATM at S1981, ATR at S428, and CHK1 at S345 and S317 significantly decreased after radiation-induced DNA damage (Fig. 4E). Conversely, the overexpression of PREX2 augmented the activation of the DNA-PKcs and ATM signaling pathway. To validate these results, PREX2-overexpressed RKO cells were treated with the PREX2 inhibitor P-Rex inhibitor 1 (PREX-in1), which blocks the Rac-GEF activity of full-length PREX2 in vitro at low micromolar concentration without affecting the activities of several other Rho-GEFs [27]. PREX-in1 effectively nullified the impact of PREX2 overexpression (Additional file 7: Fig. S3A-B).

Bioinformatics analysis on TCGA datasets unveiled a robust positive correlation between high PREX2 expression levels and key non-homologous end-joining (NHEJ) genes, including XRCC6 (Ku80), XRCC5 (Ku70), LIG4, PARP1, PRKDC (DNA-PKcs), and TOPBP1 markers (Fig. 4F, Additional file 7: Fig. S3C). Simultaneously, there are a strong positive correlation was observed between high PREX2 expression levels and key microhomology-mediated end-joining (MMEJ) genes or key homologous recombination (HR) genes (Additional file 7: Fig. S3C). These data signify that PREX2 overexpressing cells exhibit a heightened capacity for DNA repair.

### PREX2 silences the cGAS/STING/IFNs signaling pathway by inhibiting cytosolic dsDNA accumulation

**Fig. 5:**
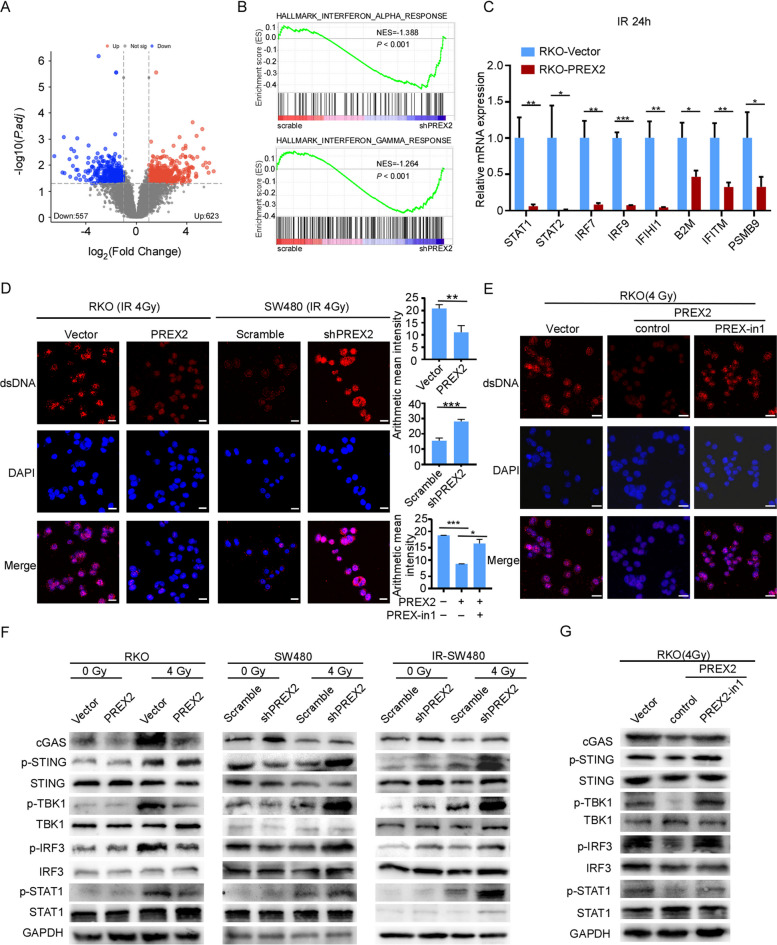
PREX2 deficiency promotes cytosolic dsDNA accumulation to activate STING signaling. **A** Volcanic maps showed significant differential genes after RNA-seq screening. **B** GSEA analyses in RNA-seq data. **C** qRT-PCR detection of the expression levels of IFN-I response genes in PREX2 overexpressed RKO cells at 24 h after 4 Gy radiation. **D** Immunofluorescence assay detection of the dsDNA in PREX2 overexpressed RKO cells and PREX2-knockdown SW480 cells after 4 Gy radiation. Scale bar: 20 μm. **E** Immunofluorescence assay detection of the dsDNA in PREX2 overexpressed RKO cells treated with or without PREX-in1 at 24 h after 4Gy radiation. **F** Western blot analysis detection of the expression of cGAS, STING, TBK1, STAT1, and IRF3 and the phosphorylation of STING, TBK1, STAT1, and IRF3 in stable overexpression and interference cell lines with or without 4 Gy radiation. **G** Western blot analysis detection of the expression of cGAS, p-STING, STING, p-TBK1, TBK1, p-STAT1, STAT1, p-IRF3, and IRF3 in PREX2 overexpressed RKO cells treated with or without PREX-in1 at 24 h after 4Gy radiation. *p < 0.05, **p < 0.01, ***p < 0.001

To unravel the mechanism through which PREX2 promotes radioresistance in CRC, we conducted RNA-seq on IR-SW480 cells with or without PREX2 knockdown. The analysis revealed that 623 upregulated and 557 downregulated genes in PREX2 knockdown IR-SW480 cells compared to the control groups (Fig. 5A, Additional file 8: Table S5). Gene Set Enrichment Analysis (GSEA) was employed to scrutinize the PREX2-regulated gene signatures (Additional file 9: Table S6). According to the GSEA results, lower PREX2 expression positively correlated with the enrichment of interferon-alpha and interferon-gamma responses (Fig. 5B).

Consistent with these findings, PREX2 overexpression significantly suppressed genes relevant to the type I interferon (IFN) response, including STAT1 (signal transducer and activator of transcription 1), STAT2 (signal transducer and activator of transcription 2 ), IRF7 (interferon regulatory factor 7), IRF9 (interferon regulatory factor 9), IFIH1 (interferon induced with helicase C domain 1), B2M (beta-2-microglobulin ), IFITM (interferon-inducible transmembrane), and PSMB9 (proteasome 20S subunit beta 9) in RKO cells at 24 h after 4 Gy radiation (Fig. 5C).

Previous studies have demonstrated that the type I IFN response is modulated by cGAS bound to dsDNA, activating the cGAS/STING pathway in cells exposed to radiation [10]. Consequently, we investigated cytosolic dsDNA using confocal microscopy. The results indicated that PREX2 silencing increased the dsDNA accumulation in the cytosol, while PREX2 overexpression decreased dsDNA accumulation at 24 h after 4 Gy radiation (Fig. 5D). Furthermore, treating PREX2 overexpressed RKO cells with PREX-in1 nullified the effect of PREX2 overexpression on dsDNA accumulation in the cytosol (Fig. 5E).

Subsequently, we examined whether PREX2 influences the cGAS/STING pathway. PREX2 silencing enhanced the phosphorylation of STING, IRF3 (interferon regulatory factor 3), TBK1 (TANK-binding kinase 1), and STAT1 and increased the expression of cGAS in cells at 24 h after 4 Gy irradiation. In contrast, PREX2 overexpression decreased these levels (Fig. 5F). Similar results were obtained when PREX overexpressed RKO cells were treated with PREX-in1 (Fig. 5G). These findings suggest the upregulation of the cGAS/STING/IFNs pathway by knocking down PREX2 after radiation.

### PREX2 modulates radiation-induced immunogenic cell death and CD8+ T cell infiltration in CRC by suppressing IFNs-I

**Fig. 6:**
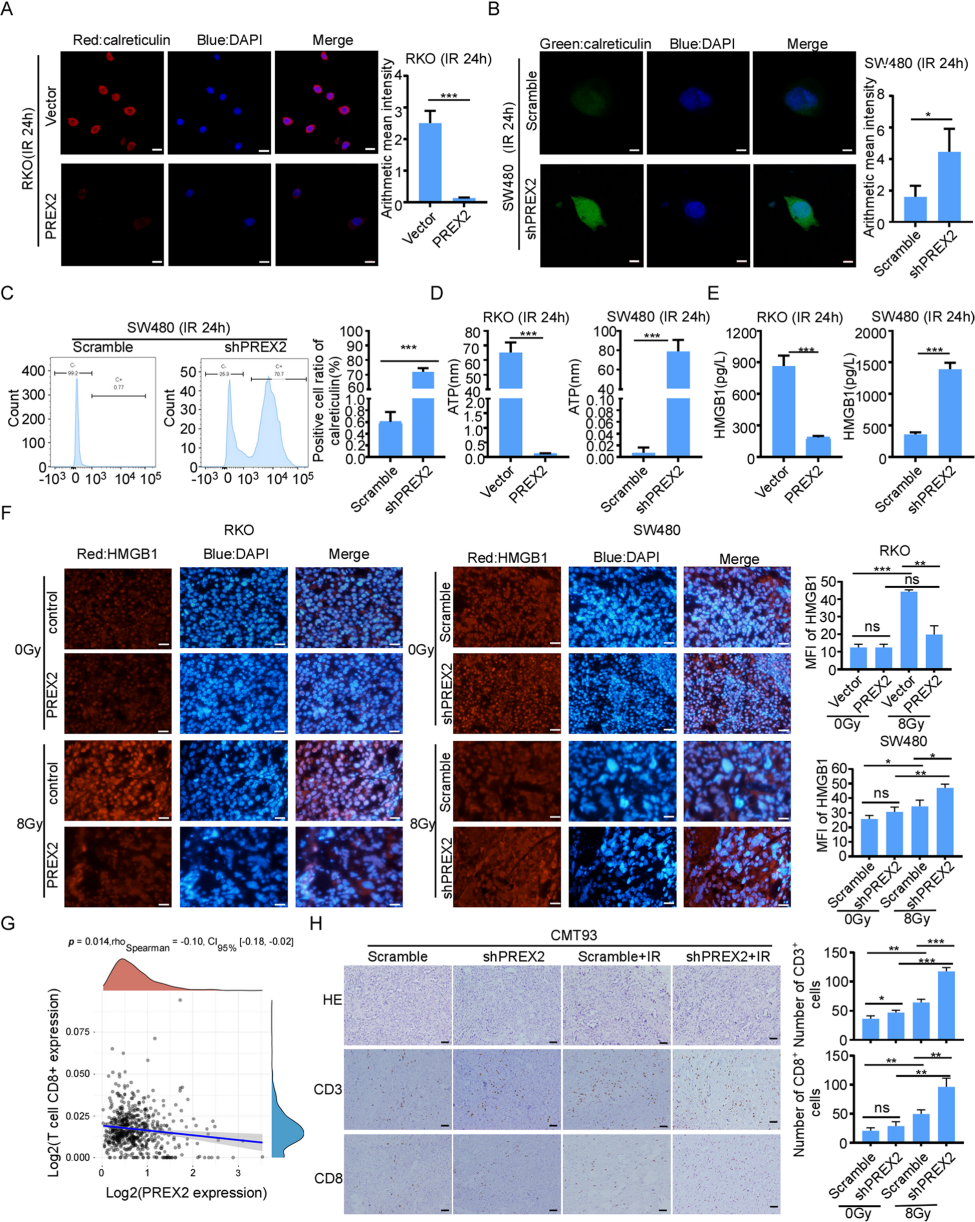
PREX2 inhibited radiation-induced immunogenic cell death and CD8^+^ T cell infiltration. **A** Immunofluorescence assay detection of the calreticulin (CRT) expression in PREX2 overexpressed RKO cells at 24 h after 4 Gy radiation. Scale bar: 20 μm. **B** Immunofluorescence assay detection of the CRT expression in PREX2 knocked down SW480 cells at 24 h after 4 Gy radiation. Scale bar: 10 μm. **C** Flow cytometry analysis confirmed that overexpressing PREX2 reduced CRT protein levels. **D** Detection of adenosine triphosphate (ATP) secretion by luciferin-based ATP assay kit. **E** Detection of high mobility group protein B1 (HMGB1) release by ELISA kit. **F** The representative images of HMGB1 immunofluorescence staining of xenograft tumors were shown (left). Scale bar: 10 μm. Quantification of HMGB1 means fluorescence intensity was analyzed (right). **G** The correlations between PREX2 expression and the immune score of CD8^+^ T cells were analyzed by the EPIC algorithm in TCGA-CRC. **H** Representative HE images and IHC images of CD3 and CD8 staining (left) and statistical analysis (right) of CMT93 tumors. Scale bar: 100 μm. ns, not significant, *p < 0.05, **p < 0.01, ***p < 0.001

Radiation holds the potential to induce immunogenic cell death, enhancing radiosensitivity in tumor cells [28]. In Fig. 6A–C, RKO cells overexpressing PREX2 and treated with 4 Gy radiation displayed fewer cell-surface CRT exposure, while PREX2 knockdown tumor cells exhibited significantly more CRT exposure. Moreover, compared to control groups, 4 Gy radiation treatment significantly increased ATP release from PREX2 knockdown SW480 cells and decreased ATP secretion from PREX2 overexpressing RKO cells (Fig. 6D). Upon cellular necrosis or damage, the nuclear protein HMGB1 is released. HMGB1 content in the cell culture supernatant 24 h after 4 Gy radiation, as measured ELISA, showed increased secretion in PREX2-knockdown CRC cells and decreased secretion in PREX2-overexpressing RKO cells (Fig. 6E). Immunofluorescence and IHC further confirmed elevated HMGB1 expression and secretion in PREX2-knockdown tumors post-IR, while PREX2 overexpression decreased HMGB1 expression and secretion in mice following radiotherapy (Fig. 6F, Additional file 10: Fig. S4A).

Additionally, PREX2 overexpression significantly suppressed IFN-I response genes in RKO cells treated with 4 Gy radiation (Fig. 5C). These results indicate that PREX2 knockdown induces potent immunogenicity. GO analysis revealed that DEGs in PREX2 knockdown IR-SW480 cells, compared to control groups, were significantly enriched in the regulation of leukocyte migration, cell chemotaxis, and leukocyte chemotaxis (Additional file 10: Fig. S4B). Using the EPIC algorithm to analyze the correlation between PREX2 gene expression and immune score in TCGA data, we found that PREX2 expression had a close negative correlation with CD8^+^immune cells (Fig. 6G, Additional file 10: Fig. S4C). Moreover, IHC staining demonstrated a significant increase in CD3^+^ and CD8^+^ cells in PREX2 knockdown CMT93 tumors in irradiated mice (Fig. 6H). These findings suggest that PREX2 may influence the infiltration of CD8^+^ T cells into the tumor microenvironment.

### PREX-in1 enhances the efficacy of IR therapy in CRC mouse models

**Fig. 7:**
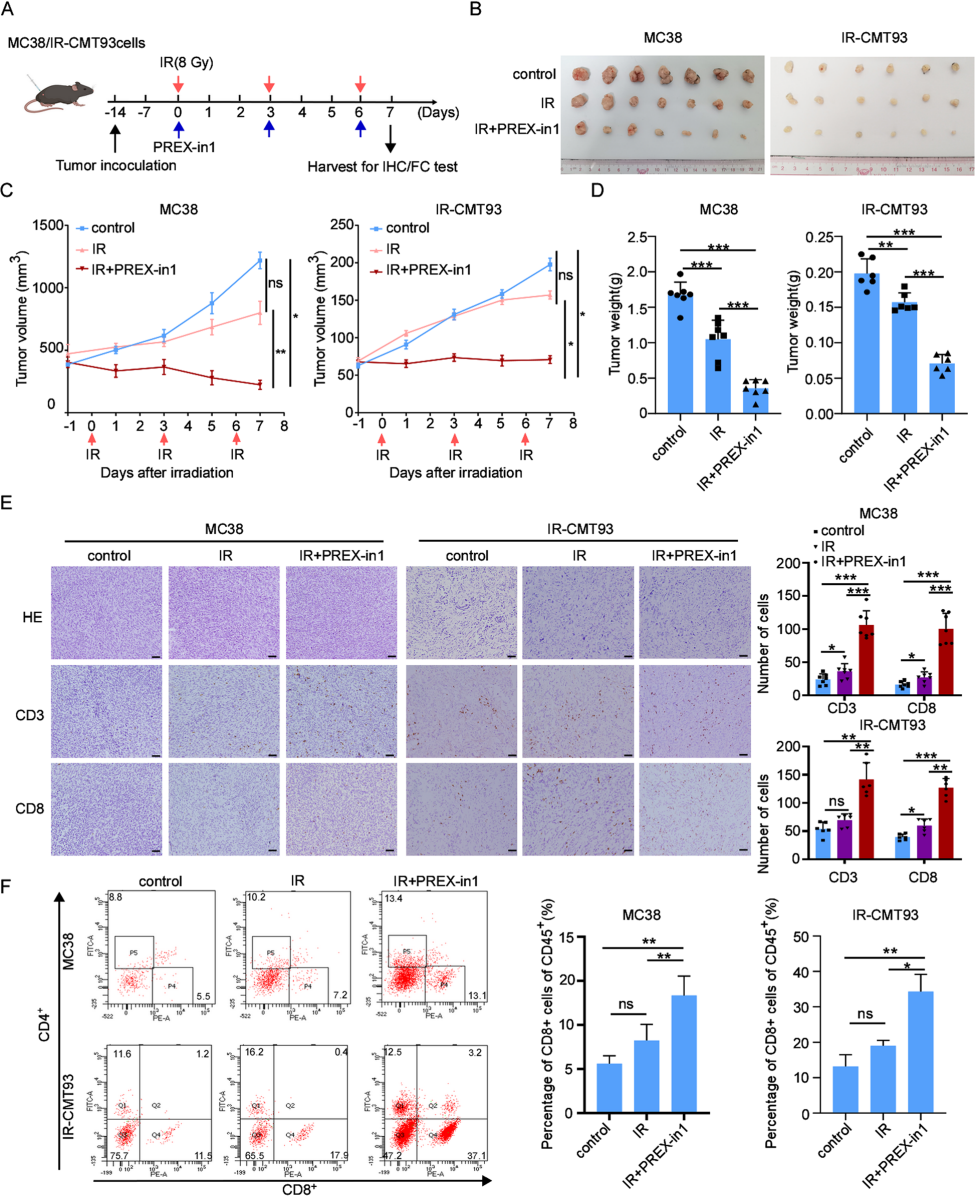
PREX-in1 sensitizes the efficacy of IR therapy in CRC mouse models. **A** The overall design of the animal experiments. **B** The representative image of MC38 and IR-resistant cell line IR-CMT93 tumors treated with IR or IR+PREX-in1. **C**, **D** The volume curve and weight of MC38 and IR-CMT93 tumors treated with IR or IR+PREX-in1. **E** Representative HE images and IHC images of CD3 and CD8 staining (left) and statistical analysis (right) of MC38 and IR-CMT93 tumors. Scale bar: 100 μm. **F** Representative flow cytometry images (left) and quantitative analysis (right) of tumor-infiltrating lymphocytes in MC38 and IR-CMT93 tumors. ns, not significant, *p < 0.05, **p < 0.01, ***p < 0.001

The aforementioned results indicate that PREX2 promotes resistance to radiation treatment in CRC cells, suggesting that inhibiting PREX2 may increase sensitivity to IR in CRC. To assess the function of PREX-in1 in IR resistance of CRC, we established a tumor homologous transplantation mouse model by subcutaneously injecting of MC38 and IR-resistant CMT93 (IR-CMT93) cells and evaluated the tumor microenvironment after PREX-in1 treatment (Fig. 7A). In Fig. 7B–D, compared to IR alone, the combination of IR and PREX-in1 (IR+PREX-in1) exhibited significantly reduced tumor growth. IHC staining demonstrated a significant increase in CD3^+^ and CD8^+^ cells in both MC38 and IR-CMT93 tumors in mice treated with IR+PREX-in1 (Fig. 7E). These results were corroborated by flow cytometry analysis, with combination therapy showing the highest proportion of CD45^+^ CD8^+^ CD4^-^ T cells (Fig. 7F). Furthermore, we observed consistent changes in the expression of γH2AX and the expression and secretion of HMGB1 in mice treated with IR+PREX-in1, demonstrating that PREX-in1 significantly promotes radiation-induced DNA damage and immunogenic cell death in CRC (Additional file 10: Fig. S4D). Taken together, IR+PREX-in1 elicited a favorable immune profile by enhancing CD8^+^ T cell infiltration in CRC.

## Discussion

This study establishes, for the first time, a direct correlation between PREX2 expression and the radiation response in colorectal cancer (CRC). Comparative analysis reveals elevated PREX2 expression in radioresistant CRC tissues compared to radiosensitive counterparts. High PREX2 levels correlate with TRG classification and shorter progression-free survival in CRC patients. Furthermore, PREX2 overexpression significantly augments the radioresistance of CRC cells both in vitro and in vivo. Mechanistically, PREX2 promotes DNA repair by upregulating DNA-PKcs, inhibiting radiation-induced immunogenic cell death, and suppressing CD8^+^ T cell infiltration through the cGAS/STING/IFNs pathway. Blocking PREX2 enhances the efficacy of IR therapy in vivo. In summary, this study not only identifies PREX2 as a potential biomarker for colorectal cancer radiation therapy response but also offers insights into a novel treatment strategy.

PREX2, previously recognized as an oncogene in diverse cancers, such as melanoma [14, 29], hepatocellular carcinoma [16], lung carcinoma [30], and pancreatic ductal adenocarcinoma [17], is a GEF regulating Rac activity under activated PI3K and G-coupled receptor signaling [31]. Additionally, PREX2 has been implicated in inhibiting PTEN activity through direct interaction of its DH-PH catalytic domain with PTEN [15, 30, 32]. However, its precise role remains elusive. In this study, we found that PREX2 expression was upregulated in radioresistant CRC, both in the array profiling from rectal cancer patients (GSE145037 and GSE150082) and in clinical CRC tissues. Low PREX2 expression correlates significantly with favorable clinicopathological parameters and good prognosis. PREX2 overexpression compromises the radiosensitivity of CRC cells, suggesting its potential as a biomarker for radiation resistance in CRC.

The primary mechanism of tumor radiotherapy involves inducing DNA damage. To elucidate the impact of PREX2 on DNA damage repair, we find that PREX2 silencing activates of γH2AX in SW480 cells. Furthermore, PREX2 promotes DNA double-strand break repair by upregulating DNA-PKcs expression and activating the ATR pathway. Bioinformatics results indicate PREX2 association with NHEJ signaling pathways. DNA-PKcs, known to mediate NHEJ, represents an imprecise yet rapid DNA repair method that occurs throughout the cell cycle, connecting only the two ends of the DSB and without restoring the original DNA sequence [33–35]. In this study, the correlation of PREX2 with DNA-PKcs suggests its involvement in the NHEJ pathway. However, bioinformatics results also link PREX2 to MMEJ and HR signaling pathways, indicating potential contributions from other repair pathways in the DDR process.

To delve deeper into the intricate mechanism of PREX2 in colorectal cancer (CRC) radioresistance, we conducted RNA-seq to unveil and identify pathways influenced by PREX2 in CRC cells. Notably, the differentially expressed genes (DEGs) triggered by PREX2 knockdown exhibited significant enrichment in the regulation of leukocyte migration, cell chemotaxis, and leukocyte chemotaxis. These findings strongly suggest the involvement of PREX2 in the immune response provoked by radiotherapy in tumor cells. Subsequent analysis unveiled a positive correlation between lower PREX2 expression and the enrichment of interferon-alpha and interferon-gamma responses. Existing research highlights the significance of the cytoplasmic DNA sensing-mediated cGAS/STING signaling pathway in triggering an active type I IFN response [36]. Our study illustrates that PREX2 silencing prompts the accumulation of double-strand break (DSB) fragments in the cytosol. Numerous studies emphasize the critical role of the cGAS/STING pathway in DNA damage sensing, demonstrated in lung cancer [37], pancreatic cancer [38], and CRC [39]. In line with this, our study reveals that PREX2 knockdown activates the cGAS/STING signaling pathway, significantly intensifying the phosphorylation of STING, IRF3, TBK1, and STAT1, along with an increase in cGAS expression 24 h post-exposure to 4 Gy radiation. Furthermore, our investigations indicate that PREX2 serves as a pivotal regulator of radiation-induced immunogenic cell death (ICD), inhibiting exposure of calreticulin (CRT) on the cell surface, suppressing HMGB1 and ATP release, and impeding interferon-I (IFN-I) production in CRC cells exposed to 4 Gy radiation. Prior studies have established the immunostimulatory effects of radiation to be associated with DNA damage response, mediated through canonical pathways regulating autoimmunity and response to viral infections [37, 40]. Our research substantiates the crucial role of that type I IFNs in bridging DNA damage in tumor cells and antitumor adaptive immunity post-radiation. These findings affirm that silencing of PREX2 activates the cGAS/STING/IFNs pathway following radiation, potentially predicting the tumor response to combinations of radiation and immune checkpoint inhibitors.

In conclusion, our data highlight the potential of PREX2 as a therapeutic target. Targeting PREX2 with the small molecule PREX-in1 significantly enhances the efficacy of CRC radiotherapy. Previous studies have unveiled the intrinsic activation of the type I IFN pathway by cancer cells and the production of IFNβ by cancer cells as crucial mechanisms for radiation-induced antitumor T cells, essential for eliciting anti-tumor effectors mediating abscopal responses [40]. In line with this discovery, our study demonstrates that the combination of IR+PREX-in1 induces a favorable immune profile by augmenting CD8^+^ T-cell infiltration in CRC. Thus, PREX2 emerges as an appealing target for overcoming radioresistance in CRC [41, 42].

## Conclusions

**Fig. 8:**
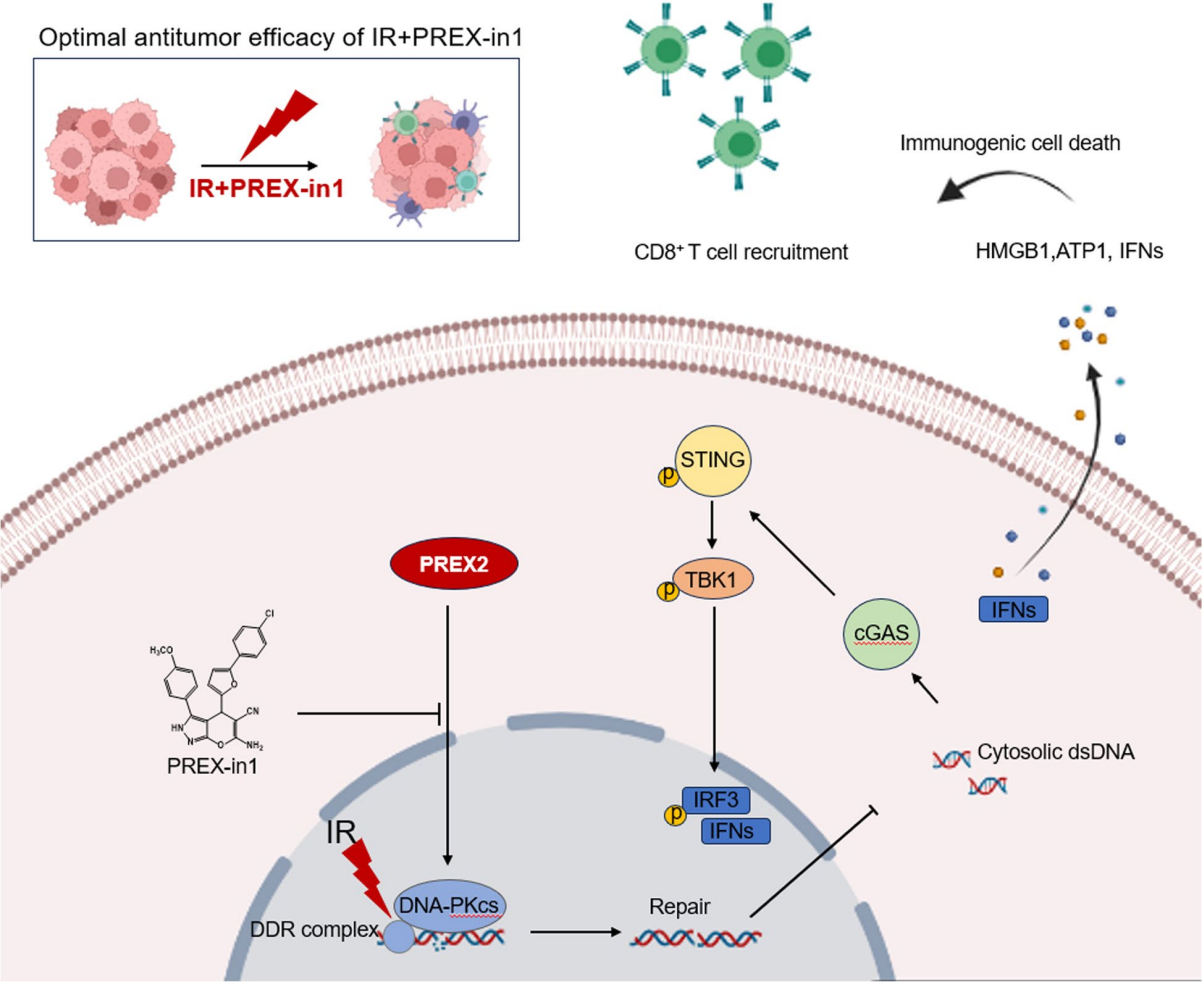
A proposed model for illustrating the function and mechanism of PREX2 in CRC radiosensitization

In summary, our discoveries underscore the pivotal role of PREX2 in colorectal cancer (CRC) radiation resistance, with its influence predominantly channeled through the cGAS/STING/IFN signaling pathway. Moreover, the inhibition of PREX2 substantially enhanced the effectiveness of ionizing radiation (IR) therapy in vivo (Fig. 8). These revelations posit PREX2 as a potential biomarker for radioresistance and a promising therapeutic target to successfully surmount radioresistance in CRC.

## Supplementary Information

**Additional file 1:** **Table S1.** Sequences of the shRNAs and primers used in the study.

**Additional file 2:** **Table S2.** List of antibodies used in this study.

**Additional file 3:** **Fig. S1.** Construction of IR-resistant SW480. (A) The proposed model for constructing IR-resistant SW480 cell lines. (B) The radiosensitivity of IR-resistant SW480 cells and parental cells was detected using a radiation clonogenic survival assay and calculated by survival fraction using the multi-target single-hit model. (C) The comet assay of IR-resistant SW480 cell lines after IR treatment and the statistics result of quantification of the tail moment. Scale bar:100 μm **p < 0.01, ***p < 0.001.

**Additional file 4:** **Table S3.** List of genes differently expressed in radioresistance cell line IR-SW480 compared with SW480 (|logFC|>1, adj. P.Val<0.05).

**Additional file 5:** **Table S4.** Correlation between the clinicopathological features and expression of PREX2.

**Additional file 6:** **Fig. S2.** PREX2 inhibits radiosensitization in CRC. (A) Detection of PREX2 levels by qRT-PCR in PREX2-overexpressed RKO cells and PREX2 knocked down SW480 and IR-SW480 cells. (B) Radiation clonogenic assays were performed in RKO cells overexpressing PREX2 and SW480/IR-SW480 cells with PREX2 knockdown after an increased dose of IR treatment (0, 2, 4, 6, and 8 Gy). (C) Flow cytometry was performed to detect apoptosis in IR-SW480 cells, with or without treatment with 4 Gy radiation. (D) Cell cycle progression of IR-SW480 cells treated with or without treatment with 4 Gy radiation was analyzed by flow cytometry. (E-G) Subcutaneous tumor formation in nude mice was established with PREX2- knockdown or control SW480 cells and treated with or without IR (*n* = 3/group). (E) Representative images of each group were photographed at the end of the experiment. (F-G) The volume and weight of tumors were monitored. Data were shown as means ± SEM.(H) Detection of PREX2 levels by Western blotting and qRT-PCR in PREX2 knocked down CMT93 cells. ns not significant, *p < 0.05, **p < 0.01, ***p < 0.001.

**Additional file 7:** **Fig. S3.** PREX2 promoted radiation-induced DNA damage response. (A) Representative fluorescence images of γH2AX staining in PREX2 overexpressed RKO cells treated with or without PREX-in1 at 2h after 4Gy radiation. Scale bar: 10 μm. (B) Western blot detection of the expression levels of DNA-PKc, p-DNA-PKc, p-ATM, ATM, p-ATR, ATR, p-CHK1, and CHK1 in PREX2 overexpressed RKO cells treated with or without PREX-in1 at 24h after 4Gy radiation. (C) Bioinformatics analysis of the correlation between PREX2 and genes related to NHEJ pathway (XRCC6, XRCC5, LIG4, PARP1, TOPBP1 and PRKDC), MMEJ pathway (NBN,PARP1,MRE11 and LIG3) and HR pathway (RAD51C,BRCA1, BRCA2,PALB2 and BARD1) in TCGA-CRC. **p < 0.01, ***p < 0.001.

**Additional file 8:** **Table S5.** List of genes differently expressed in IR-SW480-shPREX2 compared with IR-SW480-Scramble (|logFC|>1, adj. P.Val<0.05).

**Additional file 9:** **Table S6.** Gene sets enriched in phenotype shPREX2.

**Additional file 10:** **Fig. S4.** PREX2 inhibits radiation-induced immunogenic cell death and affects the infiltration of immune cells. (A) The representative IHC images of HMGB1 in xenograft tumor. Scale bar: 100 μm. (B) GO analysis of DEGs in RNA-seq data from IR-SW480 cells with or without PREX2 knockdown. (C) The correlations between PREX2 expression and immune score were analyzed using the EPIC algorithm in TCGA-CRC. (D) The representative IHC images of γH2AX and HMGB1 expression in MC38 and IR-CMT93 tumors treated with IR or IR+PREX-in1 were shown. Scale bar: 100 μm.

## Data Availability

The raw sequence data and processed data reported in this paper have been deposited in the Genome Sequence Archive  [41] in National Genomics Data Center [42], China National Center for Bioinformation/Beijing Institute of Genomics, and Chinese Academy of Sciences (accession no. HRA006561 and OMIX005713). Other datasets generated or analyzed for this study will be made available by the corresponding author upon reasonable request.

## Abbreviations

AJCC: The American Joint Council on Cancer
ATCC: The American Type Culture Collection
ATM: Ataxia Telangiectasia Mutated
ATP: Adenosine-5′-triphosphate
ATR: ATM and Rad3-related
B2M: Beta-2-microglobulin
cGAS: Cyclic GMP-AMP synthase
CHK1: Checkpoint kinase 1
CRC: Colorectal cancer
CRT: Calreticulin
DAMPs: Damage-associated molecular patterns
DCs: Dendritic cells
DDR: DNA damage response
DEGs: Differentially expressed gene
DNA-PKcs: DNA-dependent protein kinase catalytic subunit
DSBs: Double-strand breaks
dsDNA: Double-stranded DNA
ELISA: Enzyme-linked immunosorbent assay
EPIC: The Estimating the Proportions of Immune and Cancer cell
FBS: Fetal bovine serum
GEO: The public Gene Expression Omnibus
GSEA: Gene set enrichment analysis
H2AC19: H2A clustered histone 19
HMGB1: High mobility group box 1 protein
HR: Homologous recombination
ICD: Immunogenic cell death
IFIH1: Interferon induced with helicase C domain 1
IFITM: Interferon-inducible transmembrane
IFN-I: Type I interferon
IHC: Immunohistochemical
IR: Ionizing radiation
IR+PREX-in1: The combination of IR and PREX-in1
IR-CMT93: IR-resistant CMT93
IRF7: Interferon regulatory factor 7
IRF9: Interferon regulatory factor 9
MMEJ: Microhomology-mediated end-joining
nCRT: Neoadjuvant chemoradiotherapy
NHEJ: Non-homologous end-joining
pCR: Pathological complete response
PI3K: Phosphoinositide 3-kinase
PREX2: Phosphatidylinositol-3,4,5-triphosphate-dependent Rac exchange factor 2
PREX-in1: P-Rex inhibitor 1
PSMB9: Proteasome 20S subunit beta 9
PTEN: Phosphatase and tensin homolog
RacGEFs: Rac guanine nucleotide exchange factor family
RCD: Regulatory cell death
RNA-seq: RNA-sequencing
RSI: The radiosensitivity index
RT-qPCR: Real-time quantitative reverse transcription PCR
SELENON: Selenoprotein N
SF: Surviving fraction
shRNA: Short hairpin RNA
SLC6A12: Solute carrier family 6 member 12
STAT1: Signal transducer and activator of transcription 1
STAT2: Signal transducer and activator of transcription 2
STING: Stimulator of interferon genes
TBK1: TANK-binding kinase 1
TCGA: The Cancer Genome Atlas
TILs: Tumor-infiltrating lymphocytes
TRG: Tumor regression grading system
γH2AX: Phosphorylated histone H2AX
