# Correction: PREX2 contributes to radiation resistance by inhibiting radiotherapy-induced tumor immunogenicity via cGAS/STING/IFNs pathway in colorectal cancer

**DOI:** 10.1186/s12916-025-04331-4

**Published:** 2025-08-09

**Authors:** Mingzhou Li, Jianbiao Xiao, Shasha Song, Fangyi Han, Hongling Liu, Yang Lin, Yunfei Ni, Sisi Zeng, Xin Zou, Jieqiong Wu, Feifei Wang, Shaowan Xu, You Liang, Peishuang Xu, Huirong Hong, Junfeng Qiu, Jianing Cao, Qin Zhu, Li Liang

**Affiliations:** 1 Department of Pathology, Nanfang Hospital, Southern Medical University, Guangzhou, 510515 P.R. China; 2 Department of Pathology, School of Basic Medical Sciences, Southern Medical University, Guangzhou, 510515 P.R. China; 3 Guangdong Provincial Key Laboratory of Molecular Tumor Pathology, Guangzhou, Guangdong P.R. China; 4 Jinfeng Laboratory, Chongqing, 401329 P.R. China; 5 Department of Pathology, Yantai Fushan People’s Hospital, Yantai, Shandong 265500 P.R. China; 6 Yue Bei People’s Hospital Postdoctoral Innovation Practice Base, Southern Medical University, Guangzhou, 510515 P.R. China

**Correction: BMC Medicine 22, 154 (2024)**

**https://doi.org/10.1186/s12916-024–03375-2**

The authors of the original article wish to clarify an error affecting Fig. 5G (the WB bands for IRF3).

The authors accidentally misplaced the image derived from Fig. 5F (the WB band of P-IRF3 in the SW480 group for the first three lanes), during the last typesetting process resulting in an inadvertent duplication of the image of the WB bands for P-IRF3 in SW480 group.

The authors have thoroughly reviewed their experimental data and image sources to identify the correct WB images, and the corrected Fig. 5 which now accurately represents the WB results for RKO can be viewed ahead in this correction article.

**Fig. 5:**
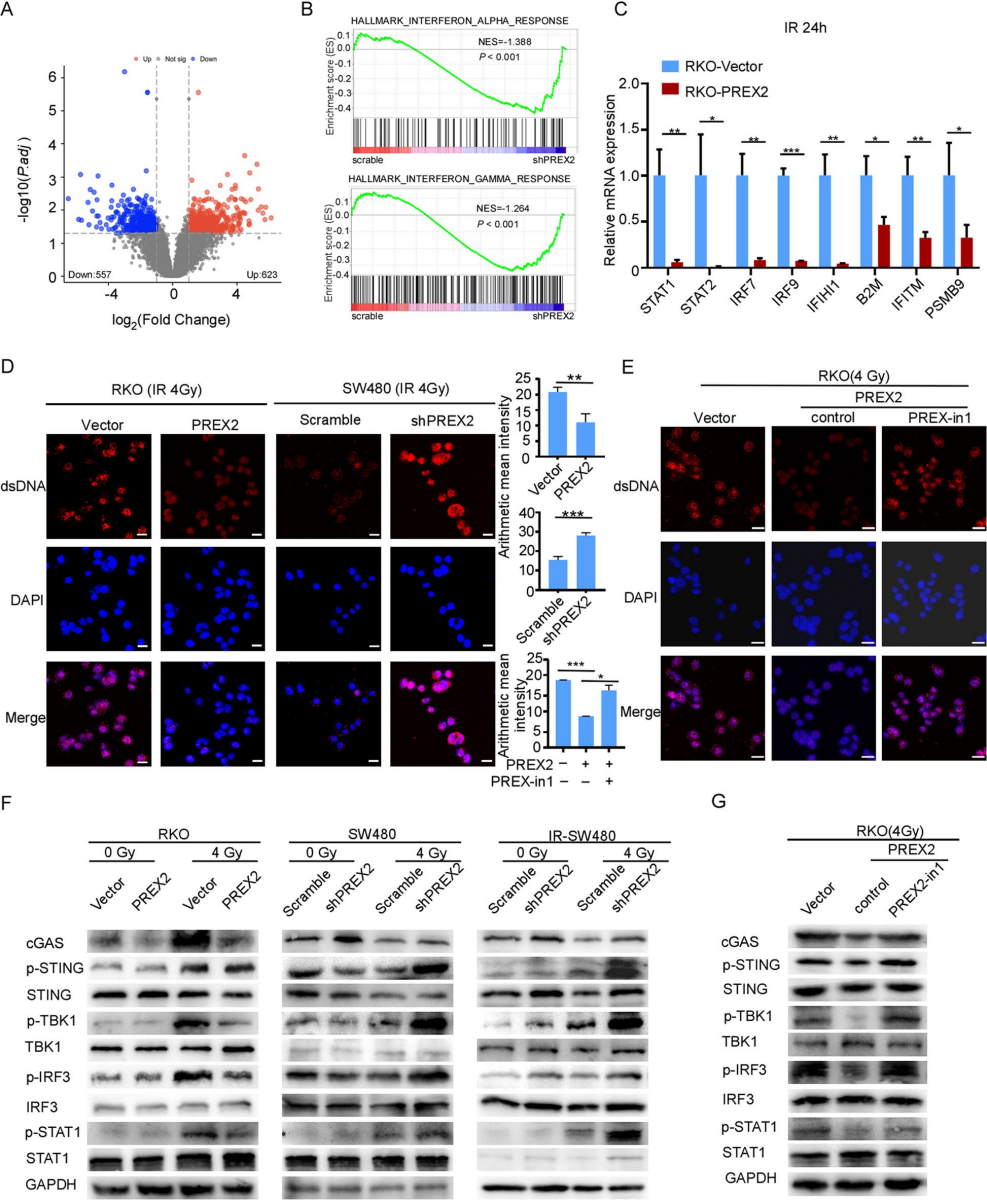
PREX2 deficiency promotes cytosolic dsDNA accumulation to activate STING signalling

