# Supplementary figures and images for: PREX2 contributes to radiation resistance by inhibiting radiotherapy-induced tumor immunogenicity via cGAS/STING/IFNs pathway in colorectal cancer
Source: BMC Med. 2024 Apr 12;22:154. doi: 10.1186/s12916-024-03375-2 (PMC11015576; doi:10.1186/s12916-024-03375-2)

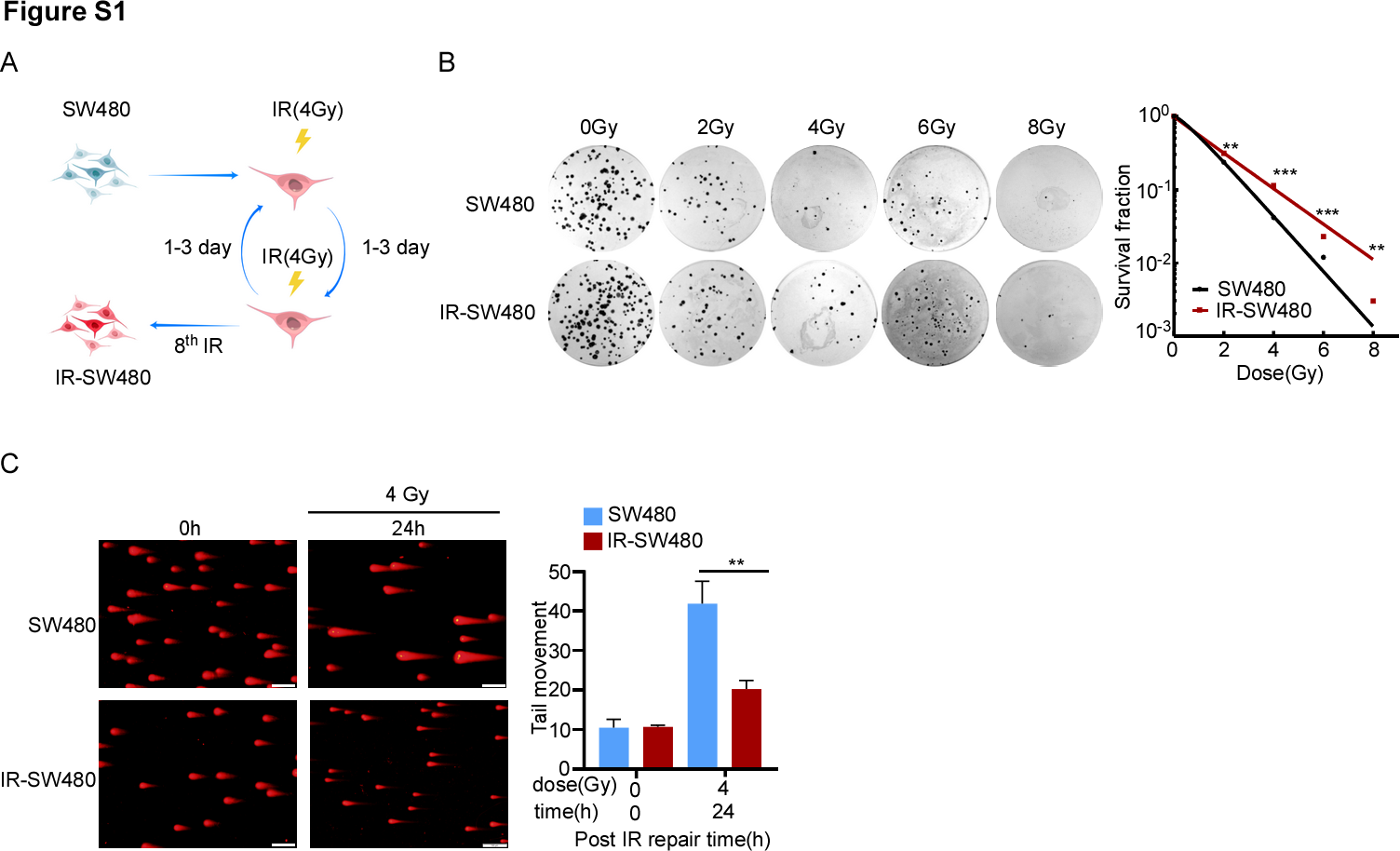

Supplement: Supplementary file 3 — Additional file 3: Fig. S1. Construction of IR-resistant SW480. (A) The proposed model for constructing IR-resistant SW480 cell lines. (B) The radiosensitivity of IR-resistant SW480 cells and parental cells was detected using a radiation clonogenic survival assay and calculated by survival fraction using the multi-target single-hit model. (C) The comet assay of IR-resistant SW480 cell lines after IR treatment and the statistics result of quantification of the tail moment. Scale bar:100 μm **p < 0.01, ***p < 0.001. [file 12916_2024_3375_MOESM3_ESM.tif]

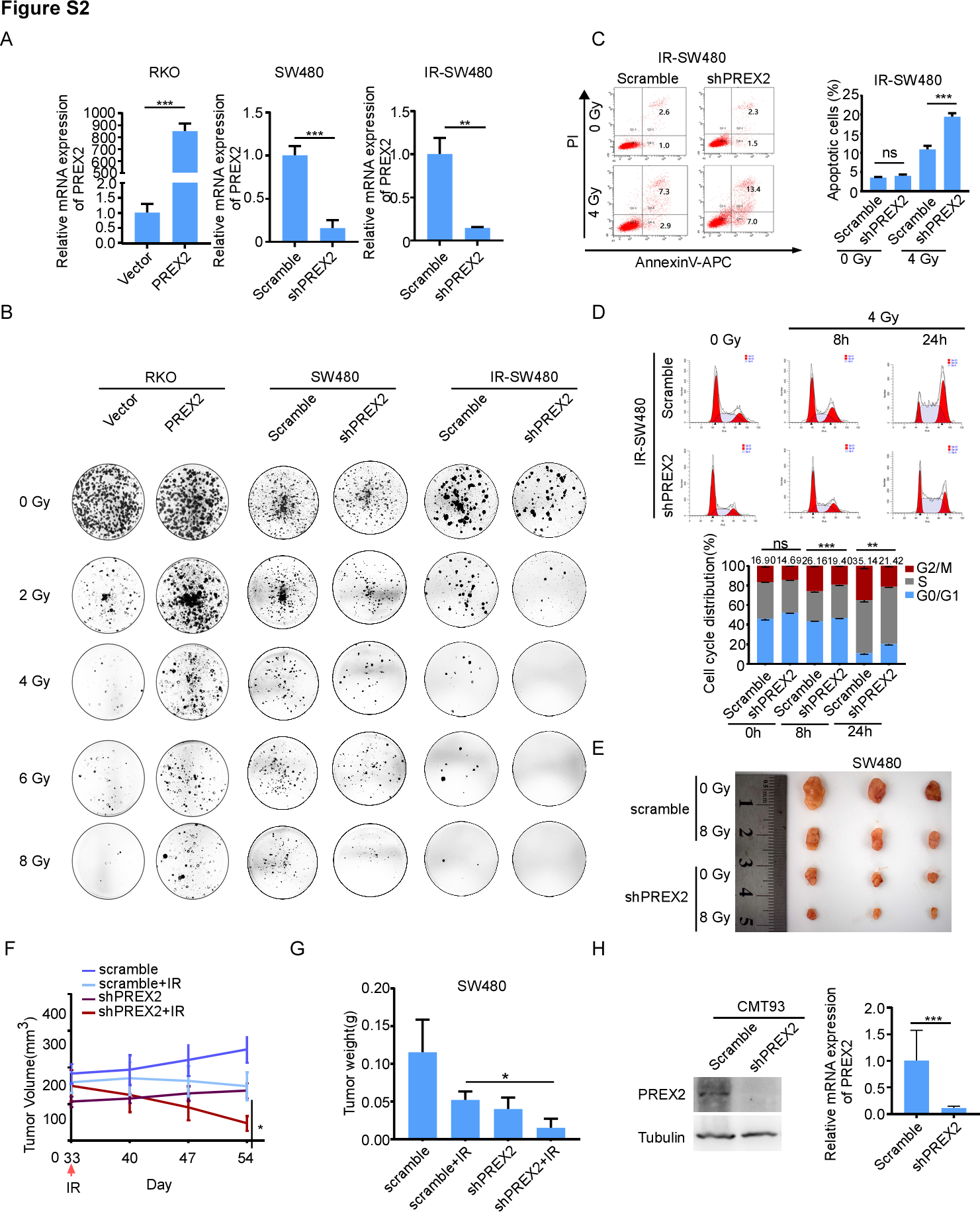

Supplement: Supplementary file 6 — Additional file 6: Fig. S2. PREX2 inhibits radiosensitization in CRC. (A) Detection of PREX2 levels by qRT-PCR in PREX2-overexpressed RKO cells and PREX2 knocked down SW480 and IR-SW480 cells. (B) Radiation clonogenic assays were performed in RKO cells overexpressing PREX2 and SW480/IR-SW480 cells with PREX2 knockdown after an increased dose of IR treatment (0, 2, 4, 6, and 8 Gy). (C) Flow cytometry was performed to detect apoptosis in IR-SW480 cells, with or without treatment with 4 Gy radiation. (D) Cell cycle progression of IR-SW480 cells treated with or without treatment with 4 Gy radiation was analyzed by flow cytometry. (E-G) Subcutaneous tumor formation in nude mice was established with PREX2- knockdown or control SW480 cells and treated with or without IR (n = 3/group). (E) Representative images of each group were photographed at the end of the experiment. (F-G) The volume and weight of tumors were monitored. Data were shown as means ± SEM.(H) Detection of PREX2 levels by Western blotting and qRT-PCR in PREX2 knocked down CMT93 cells. ns not significant, *p < 0.05, **p < 0.01, ***p < 0.001. [file 12916_2024_3375_MOESM6_ESM.tif]

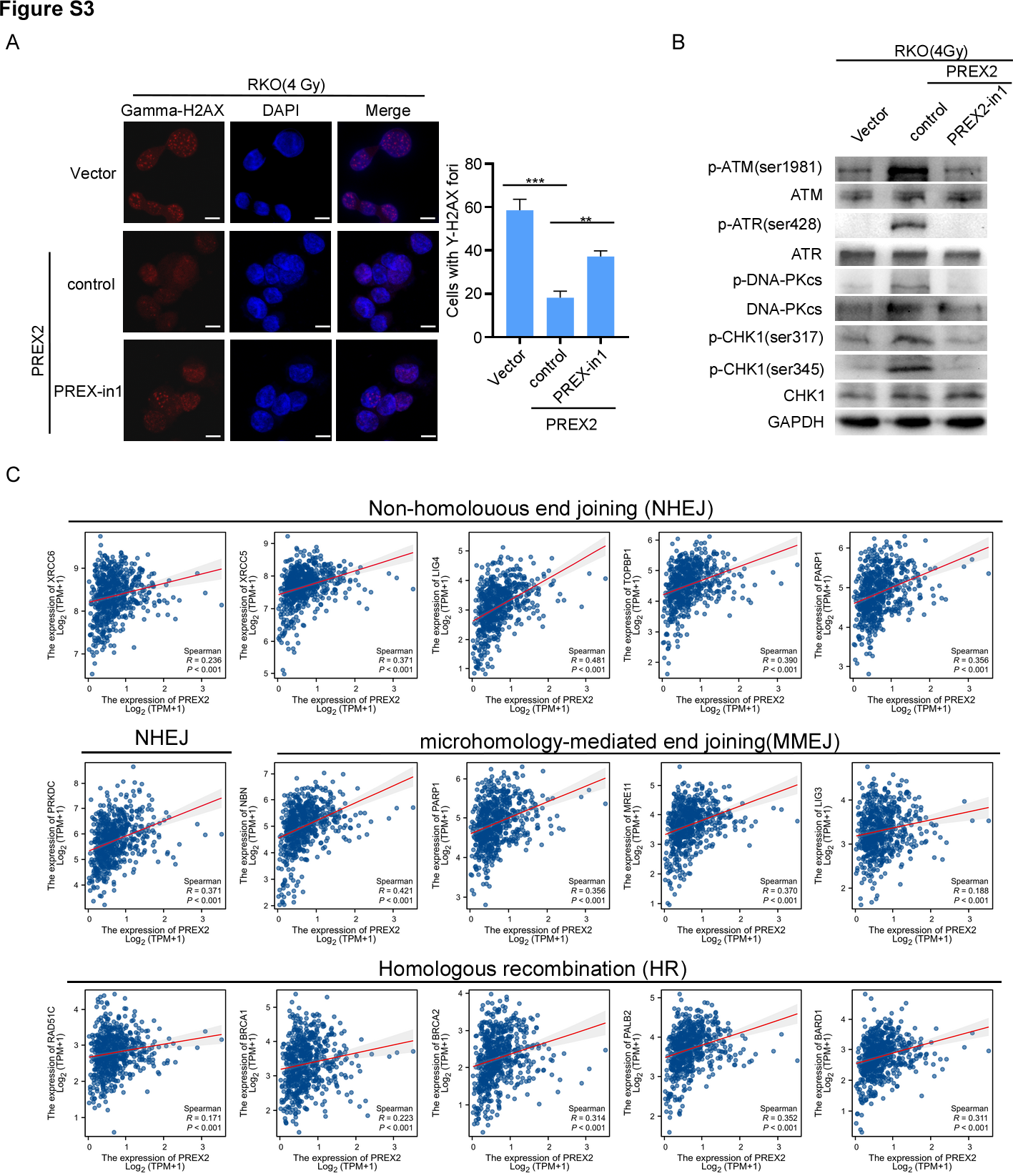

Supplement: Supplementary file 7 — Additional file 7: Fig. S3. PREX2 promoted radiation-induced DNA damage response. (A) Representative fluorescence images of γH2AX staining in PREX2 overexpressed RKO cells treated with or without PREX-in1 at 2h after 4Gy radiation. Scale bar: 10 μm. (B) Western blot detection of the expression levels of DNA-PKc, p-DNA-PKc, p-ATM, ATM, p-ATR, ATR, p-CHK1, and CHK1 in PREX2 overexpressed RKO cells treated with or without PREX-in1 at 24h after 4Gy radiation. (C) Bioinformatics analysis of the correlation between PREX2 and genes related to NHEJ pathway (XRCC6, XRCC5, LIG4, PARP1, TOPBP1 and PRKDC), MMEJ pathway (NBN,PARP1,MRE11 and LIG3) and HR pathway (RAD51C,BRCA1, BRCA2,PALB2 and BARD1) in TCGA-CRC. **p < 0.01, ***p < 0.001. [file 12916_2024_3375_MOESM7_ESM.tif]

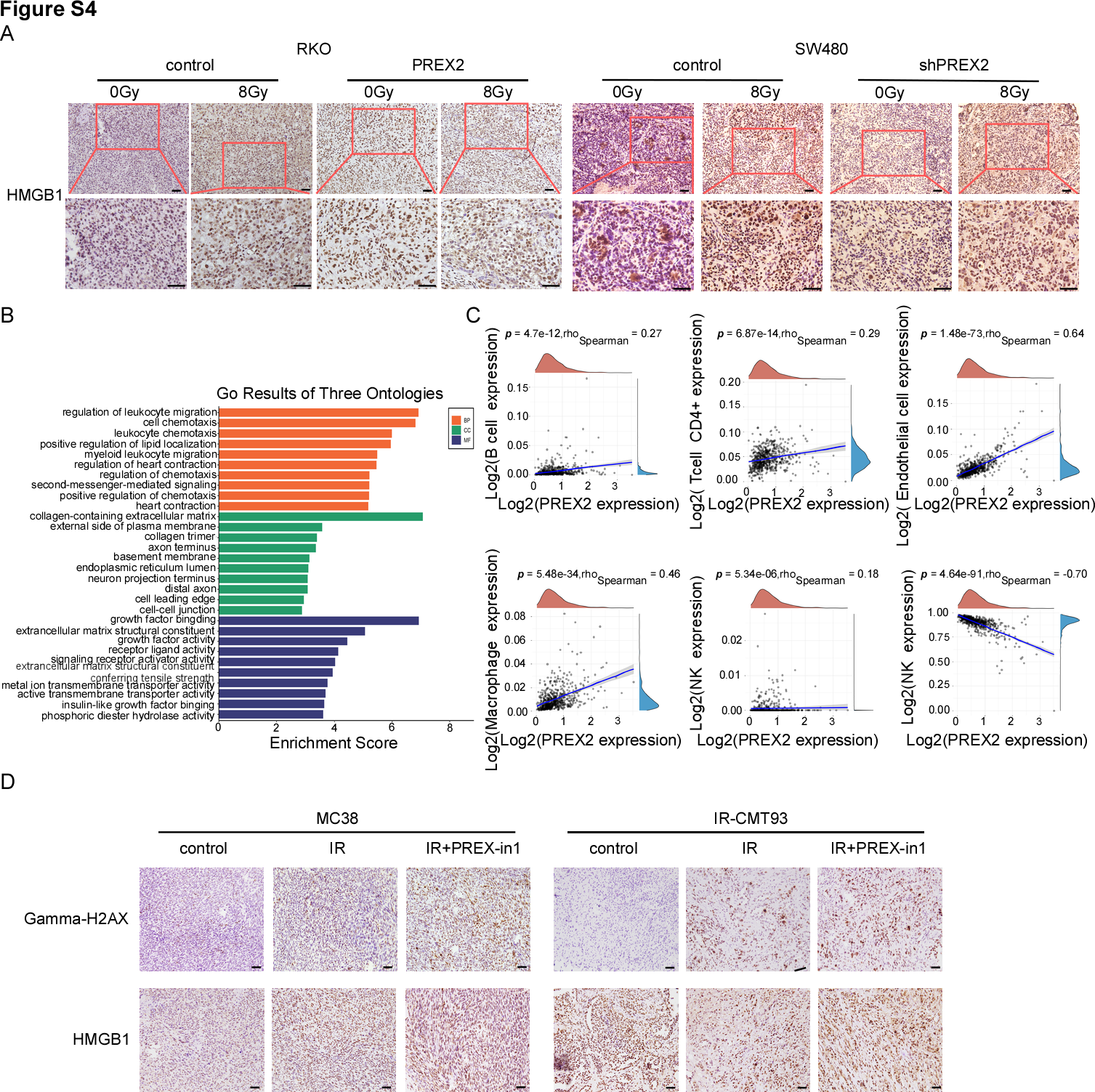

Supplement: Supplementary file 10 — Additional file 10: Fig. S4. PREX2 inhibits radiation-induced immunogenic cell death and affects the infiltration of immune cells. (A) The representative IHC images of HMGB1 in xenograft tumor. Scale bar: 100 μm. (B) GO analysis of DEGs in RNA-seq data from IR-SW480 cells with or without PREX2 knockdown. (C) The correlations between PREX2 expression and immune score were analyzed using the EPIC algorithm in TCGA-CRC. (D) The representative IHC images of γH2AX and HMGB1 expression in MC38 and IR-CMT93 tumors treated with IR or IR+PREX-in1 were shown. Scale bar: 100 μm. [file 12916_2024_3375_MOESM10_ESM.tif]
